# Thermoelectric Transport Driven by the Hilbert–Schmidt Distance

**DOI:** 10.1002/advs.202411313

**Published:** 2024-11-18

**Authors:** Chang‐geun Oh, Kun Woo Kim, Jun‐Won Rhim

**Affiliations:** ^1^ Department of Applied Physics The University of Tokyo Tokyo 113‐8656 Japan; ^2^ Department of Physics Chung‐Ang University Seoul 06974 Republic of Korea; ^3^ Department of Physics Ajou University Suwon 16499 Republic of Korea; ^4^ Research Center for Novel Epitaxial Quantum Architectures Department of Physics Seoul National University Seoul 08826 Republic of Korea

**Keywords:** quantum geometry, quantum distance, seebeck, thermoelectric power

## Abstract

The geometric characteristics of Bloch wavefunctions play crucial roles in the properties of electronic transport. Within the Boltzmann equation framework, we demonstrate that the thermoelectric performance of materials is significantly influenced by the Hilbert–Schmidt distance of Bloch wavefunctions. The connection between the distribution of quantum distance on the Fermi surface and the electronic transport scattering rate is established in the presence of magnetic and nonmagnetic impurities. The general formulation is applied to isotropic quadratic band‐touching semimetals, where one can concentrate on the role of quantum geometric effects other than the Berry curvature. It is verified that the thermoelectric power factor can be succinctly expressed in terms of the maximum quantum distance, *d*_max_. Specifically, when *d*_max_ reaches one, the power factor doubles compared to the case with trivial geometry (*d*_max_ = 0). These findings highlight the significance of quantum geometry in understanding and improving the performance of thermoelectric devices.

## Graphical abstract

Thermoelectricity, generating an electric power out of temperature differences, is shown to have a direct relationship with the Hilbert–Schmidt distance of charge and heat carriers participating the quantum transport. The performance of thermoelectric devices can be improved by engineering the geometric properties of Bloch states on the Fermi surface.

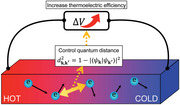

## 1 Introduction

In modern solid‐state physics, the Berry curvature^[^ ^1^, ^2^ ^]^ has played an essential role in understanding various anomalous transport phenomena^[^ ^3^, ^4^, ^5^, ^6^ ^]^ and the topological nature of solids.^[^ ^7^, ^8^, ^9^ ^]^

The Berry curvature, the imaginary part of the quantum geometric tensor, acts as an emergent magnetic field in the semiclassical equation of motion of solids,^[^ ^3^ ^]^ causing wave packets to move with an anomalous velocity proportional to the Berry curvature. This effect leads to various Hall effects, such as the anomalous Hall, valley Hall, spin Hall, and anomalous phonon Hall effects.^[^ ^5^, ^10^, ^11^, ^12^, ^13^, ^14^, ^15^, ^16^, ^17^, ^18^, ^19^ ^]^

The geometric property of the Bloch wavefunction also involves the Hilbert‐Schmidt distance, $d_{\text{HS}}^{2}\left(k,k^{\prime }\right)=1-{\left|\left\langle \psi (k)|\psi (k^{\prime })\right\rangle \right|}^{2}$, which is associated with the real part of the quantum geometric tensor when the limit $k^{\prime }\to k$ is taken.^[^ ^2^, ^20^, ^21^, ^22^ ^]^

Unlike the Berry curvature, the physical implications of the quantum metric tensor and quantum distance have only recently been explored in contexts such as charge and spin Hall effects under an inhomogeneous electric field,^[^ ^23^, ^24^, ^25^, ^26^ ^]^ current noise,^[^ ^27^ ^]^ electron‐phonon coupling,^[^ ^28^ ^]^ superfluid weight,^[^ ^29^, ^30^, ^31^, ^32^ ^]^ various magnetic responses,^[^ ^33^, ^34^, ^35^, ^36^ ^]^ and bulk‐boundary correspondence.^[^ ^37^, ^38^ ^]^

In this paper, we explore the relationship between quantum distance and thermoelectric properties, focusing on electronic contributions. Thermoelectricity, the induction of electric current by thermal gradient, has been studied extensively across various disciplines due to its possible application to the eco‐friendly energy generation.^[^ ^39^, ^40^, ^41^, ^42^, ^43^, ^44^, ^45^, ^46^, ^47^ ^]^

We begin by deriving the electronic scattering probability and disorder‐averaged transport scattering rate for a general two‐band Hamiltonian, showing that these quantities can be expressed in terms of pseudospins of the Bloch eigenvectors and, therefore, the quantum distance between Bloch states at k and $k^{\prime }$ on the Fermi surface which are not necessarily close each other.

The transport scattering rate in the presence of nonmagnetic and magnetic impurities is reduced to a simple form relying only on the maximum quantum distance in the quadratic band‐touching models, which can be applied to graphene bilayer and kagome‐like flat band systems.

Since the Berry curvature vanishes in these models, they are ideal platforms for investigating the role of the quantum distance.

Along with well‐known factors for thermoelectric performances, such as phonon scatterings and material compositions, we reveal that the geometric properties of Bloch wavefunctions, hidden within the band structures, can also play a crucial role.

## 2 The Figure of Merit in Thermoelectrics

A temperature gradient in a system induces the diffusion of charged particles, resulting in an electrical current:

(1) $$\begin{array}{c} j^{c}=-\sigma \nabla V-\sigma S\nabla T \end{array}$$

where the Seebeck coefficient (*S*) converts the temperature gradient (∇*T*) to an effective electric field. For a system in thermal equilibrium with no electric current (*j* ^*c*^ = 0) and no electric potential (∇*V* = 0), introducing a small temperature gradient δ(∇*T*) induces an electric current given by δ*j* ^*c*^ = −σ*S*δ(∇*T*).

Consequently, the change in the electric field across the device is δ(∇*V*) = −*S*δ(∇*T*), as defined by *S* = −*dV*/*dT*.

Thus, the electric power generated per unit length by the temperature gradient is:

(2) $$\begin{array}{c} \delta P_{e}=\delta j^{c}\delta \left(\nabla V\right)=\sigma S\delta \left(\nabla T\right)S\delta \left(\nabla T\right) \end{array}$$

Simultaneously, the rate of entropy production is given by

(3) $$\begin{array}{c} \frac{d}{dt}\Sigma =\frac{1}{T}\kappa \delta \left(\nabla T\right) \end{array}$$

where κ is thermal conductivity, κδ(∇*T*) is the heat flux $\dot{Q}$, and entropy change $\dot{\Sigma }=\dot{Q}/T$.

Conventionally, the figure of merit (*ZT*) and the power factor (*PF*) quantify the thermoelectric efficiency. *ZT* is defined as the ratio of the electric power to the entropy production per δ(∇*T*):

(4) $$\begin{array}{c} ZT=\frac{1}{\delta (\nabla T)}\frac{\delta P_{e}}{\dot{\Sigma }}=\frac{\sigma S^{2}T}{\kappa } \end{array}$$

Here, σ*S* ^2^ in the numerator is the power factor, and we show below that it is sensitive to the distribution of the quantum distance of Bloch wavefunctions on the Fermi surface.

Under the relaxation time approximation, the tilting of the Fermi surface due to a temperature gradient and electric field (E) is described by:

(5) $$\begin{array}{c} f\left(n,k,r\right)-f_{0}=\frac{{\left(\hbar /\tau_{nk}\right)}^{-1}}{2+2\cosh\xi }\left[eE+k_{B}T\nabla_{r}\xi \right]\cdot \nabla_{k}\xi \end{array}$$

where $\xi \equiv \left(\varepsilon_{nk}-\mu \right)/k_{B}T$,  $\tau_{nk}$ is the mean scattering time of *n*‐th band, and $f_{0}={\left(\exp\left[\left(\varepsilon_{nk}-\mu \right)/k_{B}T\right]+1\right)}^{-1}$ is the Fermi–Dirac distribution, where μ is the chemical potential. The charge and thermal current densities are

(6) $$\begin{array}{cc} j^{c} & =\frac{1}{L^{d}}\sum_{n,k}ev_{nk}\;f\left(n,k,r\right),\\ j^{th} & =\frac{1}{L^{d}}\sum_{n,k}\left(\varepsilon_{nk}-\mu \right)v_{nk}\;f\left(n,k,r\right) \end{array}$$

where *L* ^*d*^ is the volume of the system. The power factor and the figure of merit associated with electronic transport can be directly computed using Equations (5) and (6).

## 3 Quantum Distance, Pseudospin, and Transport Scattering Rate

Consider an *N*‐dimensional Hilbert space described by the eigenstates $|\psi_{n}\left(\Lambda \right)\rangle$, that smoothly depend on a set of real parameters $\Lambda =(\Lambda_{1},\Lambda_{2},\text{…})$, where *n* ∈ {1,…, *N*} denotes the band index.

The Hilbert–Schmidt distance for *n*th band is defined as follows:^[^ ^20^ ^]^

(7) $$\begin{array}{c} d_{\text{HS},n}^{2}\left(\Lambda ,\Lambda^{\prime }\right)=1-{\left|\left\langle \psi_{n}\left(\Lambda )\right|\psi_{n}(\Lambda^{\prime })\right\rangle \right|}^{2} \end{array}$$

which quantifies the distance between quantum states.

This distance reaches unity when the states are orthogonal and zero when parallel up to a global phase.

In systems with discrete translation symmetry, the crystal momentum k takes the role of Λ.

From the series expansion of the quantum distance, one can define the essential quantum geometric notions, such as quantum metric and Berry curvature, as described in Supporting Information Section I.

In a two‐band system the generic form of the Hamiltonian is given by

(8) $$\begin{array}{c} H_{k}=h_{0,k}\sigma_{0}+h_{k}\cdot \sigma \end{array}$$

where $\sigma =(\sigma_{x},\sigma_{y},\sigma_{z})$ are the Pauli matrices in the (pseudo‐)spin basis and *h*_*i*_ is a real‐valued function of momentum.

**Figure 1:**
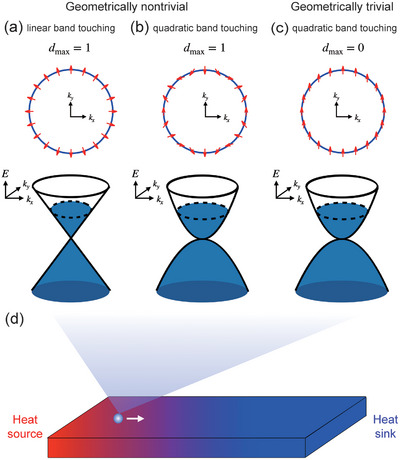
Schematic representations of the low‐energy band structure and of the pseudospins $\left(s_{y}\left(k\right),s_{z}\left(k\right)\right)$ for a) linear band‐touching system *d*_max_ = 1, b) quadratic band‐touching with *d*_max_ = 1, and c) quadratic band‐touching with *d*_max_ = 0, respectively. d) Schematic illustration of mesoscopic thermoelectric device under a temperature gradient.

The pseudospin textures visualize the geometric properties of the Bloch eigenvectors, as illustrated in **Figure** **1**a–c.

In graphene, the texture can be described by designating two of its sublattices as the pseudospin components.

Near the linear band‐touching of graphene, the average pseudospin vectors exhibit a chiral structure with winding number one, as illustrated in Figure 1a.

This winding structure is closely linked to unconventional transport properties, such as the half‐integer quantum Hall effect,^[^ ^48^, ^49^ ^]^ the absence of back‐scattering processes,^[^ ^50^, ^51^ ^]^ Klein tunneling,^[^ ^52^, ^53^ ^]^ and the weak anti‐localization phenomena.^[^ ^54^, ^55^ ^]^

Recently, it was reported^[^ ^34^ ^]^ that semimetals with a quadratic band‐touching point can exhibit a canted structure in their pseudospin texture without a quantized winding number.

Focusing on the upper band, the pseudospin on the Fermi surface is $s_{k}=\left\langle +k\right|\hat{S}\left|+k\right\rangle =h/\left|h\right|$.^[^ ^56^ ^]^ The quantum distance between states can be read out from the pseudospin structure illustrated in Figure 1a–c through the following relation:^[^ ^57^ ^]^

(9) $$\begin{array}{ccc} & & d_{{\mathrm{kk}}^{\prime }}^{2}=\frac{1}{2}\left[1-s_{k}\cdot s_{k^{\prime }}\right] \end{array}$$

indicating that $d_{{\mathrm{kk}}^{\prime }}=0$ ($d_{{\mathrm{kk}}^{\prime }}=1$) when pseudospin vectors are (anti‐)parallel.

The transport scattering rate for a state |k⟩≡|+k⟩ in the upper band, due to a perturbative potential $U_{\text{pert}}\left(r\right)$ that breaks the translation symmetry, typically from material imperfections, is given by:

(10) $$\begin{array}{c} \frac{1}{\tau_{k}}=\sum_{k^{\prime }\in \text{FS}}P_{k^{\prime }k}\left(1-\cos\theta_{k^{\prime }k}\right) \end{array}$$

where $P_{k^{\prime }k}$ represents the scattering probability per unit time, defined as:

(11) $$\begin{array}{c} P_{k^{\prime }k}=\frac{2\pi }{\hbar }{\left|\left\langle k|U_{\text{pert}}\left(r\right)|k^{\prime }\right\rangle \right|}^{2}\delta \left(\varepsilon_{k^{\prime }}-\varepsilon_{k}\right) \end{array}$$

and $\theta_{kk^{\prime }}=\cos^{-1}\left(\hat{k}\cdot \hat{k^{\prime }}\right)$ is the scattering angle, with $\hat{k}\;=\;k/\left|k\right|$.

The generic form of the scattering potential is expressed as $U_{\text{pert}}=\sum_{i=0,x,y,z}U_{i}\left(r\right)\sigma_{i}$, where it can cause both momentum transfer and a (pseudo‐)spin rotation. Employing the Fermi Golden rule, the scattering probability is

(12) $$\begin{array}{cc} P_{k^{\prime }k} & =\frac{2\pi }{\hbar }\left[(v_{q}\cdot v_{-q}-|v_{q,0}{|}^{2})\frac{1-s_{k}\cdot s_{k^{\prime }}}{2}\right.\\ & \;+\left.\sum_{i,j=0,x,y,z}\left(v_{q,i}s_{k,i}\right)\left(v_{-q,j}s_{k^{\prime },j}\right)\right]\delta \left(\varepsilon_{k^{\prime }}-\varepsilon_{k}\right) \end{array}$$

where $v_{q}$ is a four‐component vector with components given by $v_{q,i}=L^{-d}\int d^{d}r\;U_{i}\left(r\right)e^{-iq\cdot r}$ (*i* = 0, *x*, *y*, *z*), and $q=k^{\prime }-k$ is the momentum transfer. Here, we denote $s_{k,i}\equiv {\left(s_{k}\right)}_{i}$ and ${\left(s_{k}\right)}_{i=0}=1$.

This formulation shows that the scattering probability depends on the pseudospins of the eigenstates and the properties of the extrinsic scattering potential. The relation highlights the role of both intrinsic geometric properties of material and external perturbations in determining the transport characteristics.

The Fermi Golden rule, including the higher order corrections, is in Supporting Information Section VI, and one can verify that scattering probability is a function of the pseudospin and scattering potential, even when higher order corrections are included.

The distribution of the scattering potential is information specific to material and growth processes. Equation (10) and Equation (12) can be employed to compute an average transport scattering rate for any material conditions. To clarify the major relationship between the quantum distance and the scattering rate, we consider a Gaussian‐distributed scattering potential. This is to keep the primary role of disorder, breaking the translation or spin rotation symmetry of an underlying system and, at the same time, allowing further analytic manipulation. Upon averaging over the Gaussian disorder ensemble, the crossing terms containing pairs of uncorrelated scattering potential (${\left\langle v_{q,i}v_{-q,j}\right\rangle }_{i\neq j}$) vanish. Consequently, the transport scattering rate is simplified to

(13) $$\begin{array}{cc} \langle \frac{1}{\tau_{k}}\rangle =\frac{2\pi }{\hbar }\sum_{k^{\prime }\in \text{FS}} & \left[\gamma_{0}^{2}\left(1-d_{{\mathrm{kk}}^{\prime }}^{2}\right)+\frac{{\left|\gamma \right|}^{2}}{3}\left(1+d_{{\mathrm{kk}}^{\prime }}^{2}\right)\right]\\ & \times (1-\cos\theta_{kk^{\prime }}) \end{array}$$

where $\gamma_{0}^{2}=\left\langle \right|v_{q,0}{|}^{2}\rangle$ and $\gamma_{i}^{2}=\left\langle \right|v_{q,i}{|}^{2}\rangle$ are the averaged strengths of the scalar and vector components of the scattering potential, respectively.

This result explicitly shows that the distribution of the quantum distance $d_{kk^{\prime }}$ across the Fermi surface is the critical factor in determining the transport coefficients.

Specifically, the presence of nontrivial quantum geometry in a system can reduce (increase) the scattering rate when the scattering originates from spin‐independent (spin‐dependent) potentials.

In the following, we demonstrate this relationship using a 2D isotropic model. 3D examples, where we apply this geometric formula of the scattering rate, are included in Supporting Information Section IX.

## 4 Application to 2D Isotropic Band Model

We consider a 2D isotropic system, where not only the magnitude of the Fermi velocity $v_{F}=\left|\partial_{k}\varepsilon_{k}\right|$ remains constant on the Fermi surface, but also the pseudospin vector rotates uniformly with a constant angular velocity with respect to θ_*k*_ on the Fermi surface.

That is, we consider a system with spatial rotation combined with spin rotation symmetry in the Hamiltonian, $[{\hat{H}}_{0},{\hat{U}}_{\phi ,W}]=0$, where

(14) $$\begin{array}{c} {\hat{U}}_{\phi ,W}=e^{i\phi {\hat{L}}_{z}/\hbar }⊗e^{iW\phi ({\hat{s}}_{0}\cdot \sigma )/\hbar } \end{array}$$

implying that when the momentum angle θ_*k*_ changes by ϕ, the pseudospin vector rotates around ${\hat{s}}_{0}$ by an angle *W*ϕ. The cases illustrated in Figure 1a–c correspond to *W* = 1, 2, and 0, respectively. The trajectory of the pseudospin vector can be parameterized by θ_*k*_:

(15) $$\begin{array}{c} s_{k}=s_{0}+s_{⊥\alpha }\cos\left(W\theta_{k}\right)+s_{⊥\beta }\sin\left(W\theta_{k}\right) \end{array}$$

where $s_{0}$, $s_{⊥\alpha }$, and $s_{⊥\beta }$ are mutually orthogonal, $|s_{0}|=\sqrt{1-d_{\max}^{2}}$, and $|s_{⊥\alpha }\left|=\right|s_{⊥\beta }|=d_{\max}$.

**Figure 2:**
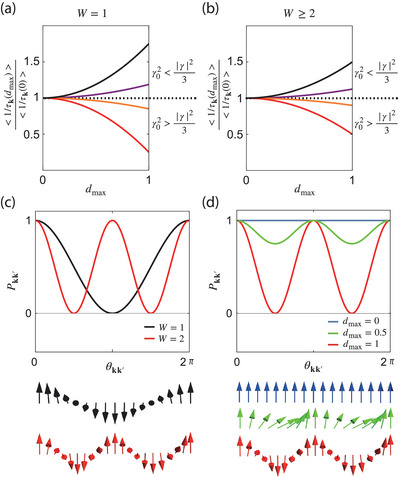
a,b) The maximum quantum distance *d*_max_ dependence of scattering rate $1/\tau_{k}$ with a) *W* = 1 and b) *W* ⩾ 2. The red, orange, purple, and black lines represent $(\gamma_{0}^{2},|\gamma {|}^{2})=c\left(1,0\right),c\left(1,2\right),c\left(1,5\right)$ and *c*(0, 1), where *c* is a positive constant. c) Scattering probability $P_{{\mathrm{kk}}^{\prime }}$ with $|k^{\prime }\left|=|k\right|$ and pseudospin structure as a function of scattering angle $\theta_{{\mathrm{kk}}^{\prime }}$ for *W* = 1 (black) and 2 (red). d) Scattering probability $P_{{\mathrm{kk}}^{\prime }}$ with $|k^{\prime }\left|=|k\right|$ and pseudospin structure as a function of scattering angle $\theta_{{\mathrm{kk}}^{\prime }}$ for the isotropic quadratic band‐touching. The blue, green, and red plots represent *d*_max_ = 0, 0.5, and 1, respectively. In (c,d), we consider γ=0 and $2\pi \gamma_{0}^{2}/\hbar =1$.

Then, the quantum distance is given by

(16) $$\begin{array}{c} d_{{\mathrm{kk}}^{\prime }}^{2}=d_{\max}^{2}\frac{1-\cos W\theta_{kk^{\prime }}}{2} \end{array}$$

where $d_{\max}=\max_{k,k^{\prime }\in \text{FS}}\left[d_{{\mathrm{kk}}^{\prime }}\right]$ represents the maximum quantum distance between all the possible pairs of Bloch eigenvectors on the Fermi surface. The scattering probability in Equation (12) is strongly influenced by *W* and *d*_max_, as shown in **Figure** **2**a,b. Averaging over the disorder ensemble, the transport scattering rate from Equation (13) becomes

(17) $$\begin{array}{cc} \langle \frac{1}{\tau_{k}^{\text{iso}}}\rangle = & \frac{2\pi }{\hbar }\frac{\rho (\varepsilon_{F})}{2}\left[\gamma_{0}^{2}\left(2-d_{\max}^{2}\right)+\frac{{\left|\gamma \right|}^{2}}{3}\left(2+d_{\max}^{2}\right)\right]\\ & +d_{\max}^{2}\frac{2\pi }{\hbar }\rho \left(\varepsilon_{F}\right)\frac{{\left|\gamma \right|}^{2}-3\gamma_{0}^{2}}{12}\delta_{W,1} \end{array}$$

where ρ(ϵ_*F*_) is the density of states at the Fermi level and the last term (∼δ_*W*, 1_) is nonzero for *W* = 1.

Details of the derivations are included in Supporting Information Section IV.

This expression shows that the transport scattering rate depends solely on the maximum quantum distance between possible scattering states and the disorder strength (γ_0_ and |γ|).

When $\gamma_{0}^{2}>{\left|\gamma \right|}^{2}/3$ ($\gamma_{0}^{2}<{\left|\gamma \right|}^{2}/3$), increasing *d*_max_ decreases (increases) the scattering rate, as shown in Figure 2a,b. Notably, the winding number *W* only impacts the scattering rate for *W* = 1.

Since the power factor is proportional to the inverse of the transport scattering rate, *PF*∝〈1/τ^iso^〉, the maximum quantum distance plays a crucial role in determining the thermoelectric performance of the isotropic model.

## 5 Application to 2D Quadratic Isotropic Band‐Touching Model

The quadratic isotropic band‐touching semimetals with symmetry ${\hat{U}}_{\phi ,W=2}$ provide an ideal platform to study the quantum distance and quantum metric. This model allows for the manipulation of the geometric structure of wavefunctions while keeping the band structure constant. Additionally, in this model, the Berry curvature is absent.^[^ ^34^, ^58^ ^]^

The model is characterized by three parameters: the mass of the upper (lower) band *m*_+_(*m*_−_) and the maximum quantum distance *d*_max_.

The Hamiltonian is given by $H_{0}\left(k\right)=h_{0}+h\cdot \sigma$, where

(18) $$\begin{array}{cc} h_{x}/\left|h\right| & =2d_{\max}\sqrt{1-d_{\max}^{2}}\sin^{2}\theta_{k},\\ h_{y}/\left|h\right| & =2d_{\max}\sin\theta_{k}\cos\theta_{k},\\ h_{z}/\left|h\right| & =(1-2d_{\max}^{2}\sin^{2}\theta_{k}) \end{array}$$

where θ_*k*_ = tan ^−1^(*k*_*y*_/*k*_*x*_) is the polar angle in momentum space and ${\left|h|=|k\right|}^{2}\left(m_{+}^{-1}-m_{-}^{-1}\right)/4$ and $h_{0}={\left|k\right|}^{2}\left(m_{+}^{-1}+m_{-}^{-1}\right)/4$ assuming $m_{+}^{-1}>m_{-}^{-1}$.

The dispersion relations for the two bands are $\varepsilon_{\pm }={\left|k\right|}^{2}/2m_{\pm }$. Thus, *d*_max_ can be controlled without altering the band structure (See Supporting Information Section II for details).

The pseudospin textures for the cases *d*_max_ = 1 and *d*_max_ = 0 are illustrated in Figure 1b,c, respectively.

The foregoing analysis clearly indicates the anti‐symmetric behavior in the transport scattering rate by pseudo‐spin independent and dependent impurities with respect to $\gamma_{0}^{2}={\left|\gamma \right|}^{2}/3$, as shown in Figure 2a,b. Let us thus focus on *U*_pert_ = *U*_0_σ_0_, i.e., |γ|=0.

In Figure 2c,d, the lower panels show the rotation of $s_{k}$ following the Fermi surface θ_*k*_ ∈ [0, 2π] for *W* = 1 (black) and *W* = 2 with *d*_max_ = 0, 0.5, 1 (blue, green, red). The scattering probability $W_{kk^{\prime }}$ plotted in Figure 2c,d oscillates *W*‐times with an amplitude proportional to the $d_{\max}^{2}$.

By integrating over states on the Fermi surface, the transport scattering rate is evaluated as

(19) $$\begin{array}{c} \langle \frac{1}{\tau_{k}^{\text{iso}}}\rangle =\frac{2\pi }{\hbar }\rho \left(\varepsilon_{F}\right)\gamma_{0}^{2}\left[\frac{2-d_{\max}^{2}}{2}-\delta_{W1}\frac{d_{\max}^{2}}{4}\right] \end{array}$$

which highlights the significant role of *d*_max_ in the transport.

Next, we consider the linear response function *L*_*ij*_ for electric and thermal currents

(20) $$\begin{array}{c} \left(\begin{array}{c} j^{c}\\ j^{th} \end{array}\right)=\left(\begin{array}{cc} L_{11} & L_{12}\\ L_{21} & L_{22} \end{array}\right)\left(\begin{array}{c} E\\ -\frac{\nabla T}{T} \end{array}\right) \end{array}$$

**Figure 3:**
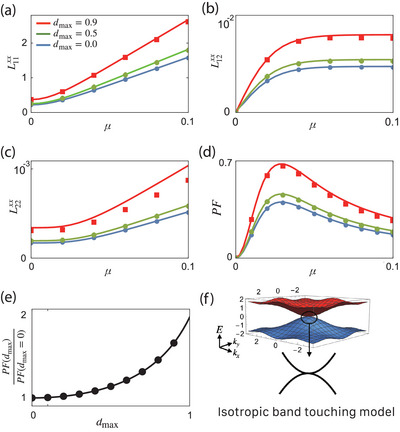
a–d) Chemical potential μ dependence of a) *L*_11_, b) *L*_12_ = *L*_21_, c) *L*_22_, and d) *PF* for *d*_max_ = 0 (blue), 0.5 (green), and 0.9 (red). e) represents the ratio between *PF*(*d*_max_) and *PF*(0). The solid lines represent the results from the isotropic quadratic touching model in Equation (18) with *m*_+_ = −*m*_−_ = 1. The dotted plots represent the results that are calculated from the lattice model Equation (22). f) Band structure of Equation (22) with *d*_max_ = 0.9. In the calculation, we fix the parameters *T* = 0.01, *k*_*B*_ = ℏ = *e* = 1, 1/τ(*d*_max_ = 0) = 0.01, and γ_0_ = *const*. For the case of Coulomb‐type impurity potential γ_0_, see Supporting Information Section VI.

In **Figure** **3**a–e (solid lines), we present the plots of *L*_*ij*_ and *PF* as functions of chemical potential for various *d*_max_ values in the 2D isotropic band‐touching model (See Supporting Information Section III for details).

While *L*_*ij*_'s are all monotonically increasing as a function of μ, *PF* exhibits a local maximum at a certain value of μ.

This implies that there is an optimal filling factor for the most efficient thermoelectric device.

The power factor can be expressed as

(21) $$\begin{array}{c} PF\left(d_{\max}\right)=\frac{2}{2-d_{\max}^{2}}PF\left(d_{\max}=0\right) \end{array}$$

This indicates that *PF* increases with *d*_max_, potentially doubling when *d*_max_ = 1 compared to the geometrically trivial case (*d*_max_ = 0), as shown in Figure 3e. This enhancement is attributed to the influence of quantum geometry on the scattering rate, highlighting that the manipulation of wavefunction geometry can significantly improve *PF*. Note that the relation in Equation (21) holds even when we consider the Coulomb‐type impurity potential (See Supporting Information Section VI).

The thermoelectric behavior experimentally observed in Bernal‐stacked bilayer graphene^[^ ^59^, ^60^ ^]^ supports our theory.

The effective Hamiltonian of the bilayer graphene belongs to the category of the 2D quadratic isotropic band‐touching model with *d*_max_ = 1.

In the studies, they demonstrated that the conventional Boltzmann transport theory works well and even becomes accurate below 130K.^[^ ^59^, ^60^ ^]^

Based on the experimental works, we extract the *PF* of the bilayer graphene as a function of the gate voltage and show that it is twice the PF of a simple (*d*_max_ = 0) parabolic band with the same effective mass, as expected from Equation (21).

Details on the thermoelectricity of the bilayer graphene are included in Supporting Information Section X.

In addition to the distribution of quantum distance on the Fermi surface, other factors also influence the performance of thermoelectric devices.

Notably, variations in chemical potential (μ) and phonon scattering are significant contributors.

The dependence on chemical potential is particularly important, as transport properties are primarily dictated by carrier charge density as well as the electronic structure near this potential.

And, the anharmonicity of lattice vibration induces phonon‐phonon scatterings that lower the phonon contribution to thermal conductivity. This will enhance the figure of merit (*ZT*) while, as we explain below, the quantum distance effect on the power factor may be weakened by electronic contribution to thermal conductivity.

It is noteworthy that changes in the geometry of electronic states also influence the electronic thermal conductivity (κ_*e*_), as shown in Supporting Information Section VIII. As a result, any increase in *PF* due to changes in scattering time will also affect κ_*e*_. However, the total thermal conductivity (κ) is not solely determined by electronic contributions. It also includes contributions from other factors such as phonons (κ_*ph*_). The geometry of electronic wavefunctions does not alter these other contributions unless there is a strong interaction between the heat carriers, such as phonons and electrons. Therefore, changing the quantum geometry of electric states to enhance *PF* will ultimately lead to a better figure of merit ($ZT=\frac{PF}{\kappa }T$), enhancing the overall efficiency of the thermoelectric material. If we consider the case where κ = κ_*e*_ + κ_*ph*_, the enhancement from controlling the geometry will have a greater impact when κ_*ph*_ is much larger than κ_*e*_. In general, phonons typically dominate heat transport in semiconductors and semimetals.^[^ ^61^, ^62^, ^63^, ^64^ ^]^ In impure metals or disordered alloys, the electron mean free path is reduced due to collisions with impurities, making the phonon contribution comparable to the electronic contribution.^[^ ^65^ ^]^ In pure metals with weak phonon‐phonon scattering, κ_*ph*_ can be comparable to, or even exceed, the electronic contribution.^[^ ^66^, ^67^ ^]^ In such materials, an increase in *PF* would significantly enhance *ZT*. In Supporting Information Section VIII, we discuss the effects of κ_*ph*_ on *ZT* in this model.

To verify the validity of the results obtained from the continuum model, we investigate the following lattice model: $H_{\mathrm{lat}}\left(k\right)=\sum_{\alpha =x,y,z}h_{\alpha }\left(k\right)\sigma_{\alpha }$, where

(22) $$\begin{array}{cc} h_{x}\left(k\right) & =d_{\max}\sqrt{1-d_{\max}^{2}}\left(1-\cos k_{y}\right)),\\ h_{y}\left(k\right) & =d_{\max}\sin k_{x}\sin k_{y},\\ h_{z}\left(k\right) & =2-2d_{\max}^{2}-\cos k_{x}+\left(2d_{\max}^{2}-1\right)\cos k_{y} \end{array}$$

For *d*_max_ < 1, it has a band crossing point at Γ point, as shown in Figure 3f. By taking the k‐p expansion near the Γ point, we derive the low energy effective model described in Equation (18) with $m_{+}^{-1}=1$ and $m_{-}^{-1}=-1$. As demonstrated in Figure 3 (square, diamond, and circular symbols), the response functions derived from the lattice model align well with those from the low energy effective model, with minor shifts due to lattice effects at high μ. This consistency clearly supports that the transport properties are primarily governed by *d*_max_, thereby validating the geometric transport formula based on *d*_max_.

## 6 Discussion

We establish a direct connection between the quantum distance and the electronic scattering probability rate, which are essential for the charge or heat transport.

Focusing on the electronic contributions, the maximum quantum distance *d*_max_ representing the intrinsic geometric property of a system has been shown to determine extrinsic thermoelectric transport coefficients averaged over an ensemble of disorder realizations.

While the role of quantum metric tensor in thermoelectric effects, such as nonlinear Nernst and Seebeck effect^[^ ^68^ ^]^ and thermoelectric generation of orbital magnetization^[^ ^69^ ^]^ are recently reported, our work shows that the entire distribution of Hilbert–Schmidt distances between Bloch states on the Fermi surface plays a crucial role in the determination of the scattering rate, which is often assumed to be an extrinsic factor in literature.

Recent theoretical and experimental research efforts have extensively focused on achieving a high *ZT* value.^[^ ^70^, ^71^, ^72^, ^73^, ^74^ ^]^

Most theoretical works focus on cases beyond the Boltzmann regime and can be broadly categorized into two groups: one addressing the SommerfeldBethe relation and the other exploring contributions that do not satisfy the SommerfeldBethe relation.

Recently, the conditions of the Sommerfeld‐Bethe relation have been clarified.^[^ ^75^ ^]^

On the other hand, the SB relation can fail when the phonon drag effect,^[^ ^72^ ^]^ electric flow generated by strong electron‐phonon interaction under a temperature gradient, is involved.

FeSb_2_ is a representative example.^[^ ^72^, ^73^, ^74^ ^]^

However, in this study, we focus on the effect of the quantum geometry of wavefunction on transport properties within the Boltzmann transport regime, where the SommerfeldBethe relation is applicable.

Although our findings highlight the role of quantum geometry in enhancing the power factor, it is essential to acknowledge that thermoelectric performance is also influenced by factors such as phonon contributions and temperature. (In Supporting Information Sections VII and VIII, we calculate the effects of phonon contributions and temperature on the model used in Figure 3). These factors affect transport properties and, consequently, *ZT*. Therefore, both quantum geometric properties and other factors must be carefully considered when optimizing thermoelectric materials.

Further investigations into various material systems, including experimental validations, are essential to fully explore the potential of geometric manipulation for enhancing thermoelectric efficiency.

While the geometric scattering rate formula Equation (13) can be used to investigate transport properties of any systems described by a two‐band Hamiltonian, the *d*_max_‐dependent formulas of the scattering rate Equation (17) and power factor Equation (21) can be applied to 2D materials with quadratic band‐crossing near the Fermi level, such as bilayer graphene and kagome‐like flat band materials.

However, the synthesis of materials hosting bands with controllable *d*_max_ is still elusive.

Lastly, extending this work to multi‐terminal thermoelectric devices and exploring extrinsic microscopic mechanisms for generating spin current from a quantum geometric perspective would be valuable directions for future research.

## Conflict of Interest

The authors declare no conflict of interest.

## Supporting information

Supporting Information

## Data Availability

The data that support the findings of this study are available from the corresponding author upon reasonable request.
