# Supplementary material for: Thermoelectric Transport Driven by the Hilbert–Schmidt Distance
Source: Adv Sci (Weinh). 2024 Nov 18;11(48):2411313. doi: 10.1002/advs.202411313 (PMC11672305; doi:10.1002/advs.202411313)
Supplement: Supplementary file 1 — Supporting Information [file ADVS-11-2411313-s001.pdf]

ADVANCED SCIENCE
Open Access

Supporting Information

for *Adv. Sci.*, DOI 10.1002/advs.202411313

Thermoelectric Transport Driven by the Hilbert–Schmidt Distance

Chang-geun Oh, Kun Woo Kim and Jun-Won Rhim**

Supporting Information: Thermoelectric Transport Driven by Quantum Distance

Chang-geun Oh

Department of Applied Physics, The University of Tokyo, Tokyo 113-8656, Japan

Kun Woo Kim

Department of Physics, Chung-Ang University, 06974 Seoul, Republic of Korea

Jun-Won Rhim

Department of Physics, Ajou University,
Suwon 16499, Republic of Korea and
Research Center for Novel Epitaxial Quantum Architectures,
Department of Physics, Seoul National University, Seoul 08826, Republic of Korea

Contents

I. **Quantum distance between eigenstates on the Fermi surface** 3

II. **Isotropic quadratic band-touching model** 3

III. **Boltzmann transport** 5

IV. **Disorder averaged transport scattering rates** 6

V. **Higher order corrections to the Fermi Golden rule** 7

VI. **Coulomb-type impurity potential in isotropic quadratic band-touching model** 11

VII. **Temperature dependence of transport properties** 13

VIII. **Figure of merit (ZT) and thermal conductivity from phonon** 15

IX. **Example: a 3-dimensional isotropic band-touching model** 16

X. **Transport properties in bilayer graphene** 16

XI. **Transport scattering rate for a single scattering strength** 18

References 20

I. QUANTUM DISTANCE BETWEEN EIGENSTATES ON THE FERMI SURFACE

When the crystal momentum changes $\boldsymbol{k} \rightarrow \boldsymbol{k} + \boldsymbol{dk}$, an eigenstate changes by $d|\psi\rangle = \sum_\nu |\partial_{k_\nu}\psi\rangle dk_\nu$, and the distance between the eigenstates is defined as $ds^2 = ||\psi(\boldsymbol{k}) - \psi(\boldsymbol{k} + \boldsymbol{dk})||^2 = \left\langle \sum_\mu \partial_{k_\mu}\psi \, dk_\mu \,\middle|\, \sum_\nu \partial_{k_\nu}\psi \, dk_\nu \right\rangle$. Using the quantum geometric tensor $Q_{\mu\nu} = \left\langle \partial_{k_\mu}\psi \,\middle|\, \partial_{k_\nu}\psi \right\rangle$, the distance is expressed as

$$ds^2 = \sum_{\mu\nu} Q_{\mu\nu} dk_\mu dk_\nu, \tag{1}$$

$$= \sum_{\mu\nu} \frac{1}{2}(Q_{\mu\nu} + Q_{\nu\mu}) dk_\mu dk_\nu, \tag{2}$$

$$\equiv \sum_{\mu\nu} g_{\mu\nu} dk_\mu dk_\nu, \tag{3}$$

where quantum metric tensor $g_{\mu\nu} = g_{\nu\mu}$ is introduced, and in the second line $dk_\mu dk_\nu = dk_\nu dk_\mu$ is used. That is, the quantum metric tensor is the symmetric part of the quantum geometric tensor, $g_{\mu\nu} = \frac{1}{2}(\left\langle \partial_{k_\mu}\psi \,\middle|\, \partial_{k_\nu}\psi \right\rangle + \left\langle \partial_{k_\nu}\psi \,\middle|\, \partial_{k_\mu}\psi \right\rangle) = \Re \left\langle \partial_{k_\mu}\psi \,\middle|\, \partial_{k_\nu}\psi \right\rangle$. $Q_{\mu\nu}$ is, in general, a complex number and its imaginary part is associated with the accumulation of U(1) phase when a state makes a loop around an area $dk_x dk_y$, the Berry curvature $\Omega_{\mu\nu} = 2\Im \left\langle \partial_{k_\mu}\psi \,\middle|\, \partial_{k_\nu}\psi \right\rangle$.

The Hilbert-Schmidt (HS) distance and quantum metric tensor. The HS distance between Bloch states at $\boldsymbol{k}$ and $\boldsymbol{k} + d\boldsymbol{k}$ is

$$d^2_{\text{HS}}(\boldsymbol{k}, \boldsymbol{k} + d\boldsymbol{k}) = 1 - |\langle \psi_k | \psi_{k+dk} \rangle|^2, \tag{4}$$

$$= 1 - \langle \psi_k | \psi_{k+dk} \rangle \langle \psi_{k+dk} | \psi_k \rangle, \tag{5}$$

$$\simeq 1 - \langle \psi_k | (|\psi_k\rangle + \sum_\mu |\partial_{k_\mu}\psi_k\rangle dk_\mu)(\langle \psi_k| + \sum_\nu \langle \partial_{k_\nu}\psi_k | dk_\nu) |\psi_k\rangle, \tag{6}$$

$$= \left\langle \sum_\mu \partial_{k_\mu}\psi \, dk_\mu \,\middle|\, \sum_\nu \partial_{k_\nu}\psi \, dk_\nu \right\rangle, \tag{7}$$

$$= \sum_{\mu,\nu} g_{\mu\nu} dk_\mu dk_\nu, \tag{8}$$

where in the second and third lines, we keep the terms up to the second order of dk_μ. In the fourth line $d\langle \psi_k | \psi_k \rangle = 0$ is used.

II. ISOTROPIC QUADRATIC BAND-TOUCHING MODEL

In this section, we show that the most general Hamiltonian describing the isotropic quadratic band-touching can be written with three parameters (the mass of upper/lower band $m_\pm$ and the

maximum quantum distance $d_{\rm max}$) and the eigenstates and pseudo spin vector can be expressed only in terms of $d_{\rm max}$ and $\theta_{\boldsymbol{k}}$.

The most general 2D quadratic band-touching Hamiltonian[1] is

$$\mathcal{H}(\boldsymbol{k}) = \sum_{\alpha=0,x,y,z} f_\alpha(\boldsymbol{k})\sigma_\alpha, \tag{9}$$

where $h_0(\boldsymbol{k}) = b_1 k_x^2 + b_2 k_x k_y + b_3 k_y^2$ and

$$h_x(\boldsymbol{k}) = t_6 k_y^2, \qquad h_y(\boldsymbol{k}) = t_4 k_x k_y + t_5 k_y^2, \qquad h_z(\boldsymbol{k}) = t_1 k_x^2 + t_2 k_x k_y + t_3 k_y^2, \tag{10}$$

The condition for the isotropic band-touching model is

$$h_0^2 = |\boldsymbol{k}|^4 \frac{1}{16}\left(\frac{1}{m_+} + \frac{1}{m_-}\right)^2, \quad h_x^2 + h_y^2 + h_z^2 = |\boldsymbol{k}|^4 \frac{1}{16}\left(\frac{1}{m_+} - \frac{1}{m_-}\right)^2, \tag{11}$$

such that the momentum-energy dispersion relation is $\epsilon_\pm = |\boldsymbol{k}|^2/2m_\pm$. The straightforward algebra provides that

$$t_1 = \frac{1}{4}\left(\frac{1}{m_+} - \frac{1}{m_-}\right), \; t_2 = 0, \; t_3 = \frac{1-2d_{\rm max}^2}{4}\left(\frac{1}{m_+} - \frac{1}{m_-}\right), \; t_4 = \frac{d_{\rm max}}{2}\left(\frac{1}{m_+} - \frac{1}{m_-}\right),$$
$$t_5 = 0, \; b_1 = \frac{1}{4}\left(\frac{1}{m_+} + \frac{1}{m_-}\right), \; b_2 = 0, \; b_3 = \frac{1}{4}\left(\frac{1}{m_+} + \frac{1}{m_-}\right), \tag{12}$$

where note that parameter $d_{\rm max}$ is involved in the Hamiltonian while its eigenvalues are only determined by mass $m_\pm$ and momentum. Its physical meaning, which is the maximum quantum distance, will be clear in the following section. Eigenvectors at $\boldsymbol{k} = (k\cos\theta_{\boldsymbol{k}}, k\sin\theta_{\boldsymbol{k}})$ are

$$|+,\boldsymbol{k}\rangle = \begin{pmatrix} \sqrt{1-d_{\rm max}^2}\sin\theta_{\boldsymbol{k}} - i\cos\theta_{\boldsymbol{k}} \\ d_{\rm max}\sin\theta_{\boldsymbol{k}} \end{pmatrix}, \tag{13}$$

$$|-,\boldsymbol{k}\rangle = \begin{pmatrix} -d_{\rm max}\sin\theta_{\boldsymbol{k}} \\ \sqrt{1-d_{\rm max}^2}\sin\theta_{\boldsymbol{k}} + i\cos\theta_{\boldsymbol{k}} \end{pmatrix}, \tag{14}$$

which is independent of $m_\pm$. Using the eigenvector of the conduction band, we can compute the pseudospin vector, $\boldsymbol{s} = \langle +\boldsymbol{k}|\hat{S}| + \boldsymbol{k}\rangle$,

$$\boldsymbol{s} = (d_{\rm max}\sqrt{1-d_{\rm max}^2}(1-\cos 2\theta_{\boldsymbol{k}}), -d_{\rm max}\sin 2\theta_{\boldsymbol{k}}, 1 - d_{\rm max}^2 + d_{\rm max}^2\cos 2\theta_{\boldsymbol{k}}), \tag{15}$$

which is normalized $|\boldsymbol{s}| = 1$, and the vector makes a circle around $\boldsymbol{s}_0 = (d_{\rm max}\sqrt{1-d_{\rm max}^2}, 0, 1 - d_{\rm max}^2)$ with radius $d_{\rm max}^2$. Note that the eigenstate can be generated by the SU(2) rotation around $\boldsymbol{s}_0$ axis:

$$|+,\boldsymbol{k}\rangle = e^{i\theta_k(\hat{s}_0\cdot\boldsymbol{\sigma})}\begin{pmatrix}1\\0\end{pmatrix}, \quad |-,\boldsymbol{k}\rangle = e^{i(\frac{\pi}{2}+\theta_k)(\hat{s}_0\cdot\boldsymbol{\sigma})}\begin{pmatrix}1\\0\end{pmatrix}, \tag{16}$$

where $\hat{s}_0 = (d_{\max}, 0, \sqrt{1 - d_{\max}^2})$. Because the pseudospin vector also rotates around $\hat{s}_0$, the Hamiltonian has the rotation symmetry by $\hat{U}_\theta$ mentioned in the main text with $W = 1$ for the above model.

III. BOLTZMANN TRANSPORT

When a system reaches a steady state under an electric field and temperature gradient, the distribution of electron in n at momentum k stays constant in time with scatterings back to the Fermi Dirac distribution:

$$\frac{df}{dt} = \frac{\partial f}{\partial t} + \frac{\partial f}{\partial r_i}\dot{r}_i + \frac{\partial f}{\partial k_i}\dot{k}_i = \frac{\partial f}{\partial \xi}\frac{\partial \xi}{\partial r_i}\dot{r}_i + \frac{\partial f}{\partial \xi}\frac{\partial \xi}{\partial k_i}\dot{k}_i, \tag{17}$$

where $\xi = (\epsilon_{nk} - \mu)/k_B T$ is introduced, and then $\frac{\partial \xi}{\partial r_i} = -\partial_{r_i}(\mu/T)$, $\frac{\partial \xi}{\partial k_i} = \frac{1}{k_B T}\partial_{k_i}\epsilon_{nk}$. The velocity in real space and momentum space is $\dot{r}_i = \frac{1}{\hbar}\partial_{k_i}\epsilon_{nk}$, $\dot{k}_i = eE_i/\hbar$, respectively.

$$\frac{df}{dt} = \frac{\partial f}{\partial \xi}\left[\partial_{r_i}\xi + \frac{1}{k_B T}eE_i\right]\frac{1}{\hbar}\partial_{k_i}\epsilon_{nk} = -\frac{f(n,k,r) - f_0}{\tau_{nk}}, \tag{18}$$

from which one can obtain the expression Eq. (5) in the main text. The linear response functions of transport can be extracted

$$\begin{pmatrix} j^c \\ j^{th} \end{pmatrix} = \begin{pmatrix} L_{11} & L_{12} \\ L_{21} & L_{22} \end{pmatrix} \begin{pmatrix} E \\ -\frac{\nabla T}{T} \end{pmatrix}. \tag{19}$$

combined with the electric current and energy current density in Eq. (6) in the main text, the coefficients are explicitly

$$L_{\mu\nu}^{ij} = -\frac{e^4}{V}\left(\frac{k_B T}{e}\right)^{\mu+\nu} \sum_{n,\boldsymbol{k}} \frac{(\hbar/\tau_{n\boldsymbol{k}})^{-1}}{2 + 2\cosh\xi} \frac{(\partial_{k_i}\xi)(\partial_{k_j}\xi)}{\xi^{2-\mu-\nu}}, \tag{20}$$

where $\mu, \nu = 1, 2$ is for electrical and heat conductivity, respectively. Note that $L_{12} = L_{21}$ for time-reversal symmetric materials according to Onsager's reciprocal theorem[2]. PF can be described using L_{ij} as

$$PF = \frac{1}{T^2}\frac{L_{12}^2}{L_{11}} \tag{21}$$

IV. DISORDER AVERAGED TRANSPORT SCATTERING RATES

For the most general type of scattering potential $U_{\rm pert} = \sum_{i=0,x,y,z} U_i(\boldsymbol{r})\sigma_i$ the scattering probability under the Born approximation is

$$P_{\boldsymbol{k'k}} = \frac{2\pi}{\hbar}\Big[(\boldsymbol{v_q}\cdot\boldsymbol{v_{\text{-}q}} - |v_{\boldsymbol{q},0}|^2)\frac{1-\boldsymbol{s_k}\cdot\boldsymbol{s_{k'}}}{2} + \sum_{i,j=0,x,y,z}(v_{\boldsymbol{q},i}\boldsymbol{s}_{\boldsymbol{k},i})(v_{\text{-}\boldsymbol{q},j}\boldsymbol{s}_{\boldsymbol{k'},j})\Big]\delta(\epsilon_{\boldsymbol{k'}} - \epsilon_{\boldsymbol{k}}), \quad (22)$$

where the scattering potential in the momentum space $v_{\boldsymbol{q},i} = \frac{1}{L^2}\int d^2\boldsymbol{r}\, U_i(\boldsymbol{r})e^{-i\boldsymbol{q}\cdot\boldsymbol{r}}$, the momentum transfer by a scattering $\boldsymbol{q} = \boldsymbol{k'} - \boldsymbol{k}$, $s_{\boldsymbol{k},i} \equiv (s_{\boldsymbol{k}})_i$ and $(s_{\boldsymbol{k}})_{i=0} = 1$ are introduced.

The disorder averaging:

$$\langle\cdots\rangle = \int\prod_i dU_i e^{-\frac{1}{2\gamma_i^2}\sum_i\int\frac{d^2\boldsymbol{r}}{V}U_i^2(\boldsymbol{r})}(\cdots), \quad (23)$$

where index $i = 0, x, y, z$ for non-magnetic and magnetic scattering potentials. For example,

$$\langle U_i(\boldsymbol{r})U_j(\boldsymbol{r'})\rangle = V\gamma_j^2\delta(\boldsymbol{r}-\boldsymbol{r'})\delta_{ij} \quad (24)$$

which is for the delta function correlated impurity scattering potential. (This can be extended to Coulomb scattering potential). The correlation between the Fourier-transformed scattering potential,

$$\langle v_i(\boldsymbol{q})v_j(\boldsymbol{q'})\rangle = \gamma_j^2\delta(\boldsymbol{q}+\boldsymbol{q'})\delta_{ij}, \quad (25)$$

which is used to remove the crossing terms. As a result,

$$\langle P_{\boldsymbol{k'k}}\rangle = \frac{2\pi}{\hbar}\Big[\langle\boldsymbol{v_q}\cdot\boldsymbol{v_{\text{-}q}}\rangle\frac{1-\boldsymbol{s_k}\cdot\boldsymbol{s_{k'}}}{2} + \langle|v_{\boldsymbol{q},0}|^2\rangle\frac{1+\boldsymbol{s_k}\cdot\boldsymbol{s_{k'}}}{2} + \sum_{i=x,y,z}\langle|v_{\boldsymbol{q},i}|^2\rangle\boldsymbol{s}_{\boldsymbol{k},i}\boldsymbol{s}_{\boldsymbol{k'},i}\Big]\delta(\epsilon_k - \epsilon_{k'}), \quad (26)$$

$$= \frac{2\pi}{\hbar}\Big[|\gamma|^2\frac{1-\boldsymbol{s_k}\cdot\boldsymbol{s_{k'}}}{2} + \gamma_0^2\frac{1+\boldsymbol{s_k}\cdot\boldsymbol{s_{k'}}}{2} + \sum_{i=x,y,z}\frac{|\gamma|^2}{3}\boldsymbol{s}_{\boldsymbol{k},i}\boldsymbol{s}_{\boldsymbol{k'},i}\Big]\delta(\epsilon_k - \epsilon_{k'}), \quad (27)$$

$$= \frac{2\pi}{\hbar}\Big[\frac{|\gamma|^2}{3}(1+d^2_{\boldsymbol{kk'}}) + \gamma_0^2(1-d^2_{\boldsymbol{kk'}})\Big]\delta(\epsilon_k - \epsilon_{k'}), \quad (28)$$

In the second line $\langle|v_{\boldsymbol{q},i}|^2\rangle = |\gamma^2|/3$ is used, and in the third line the quantum distance is introduced $\boldsymbol{s_k}\cdot\boldsymbol{s_{k'}} = 1 - 2d^2_{\boldsymbol{kk'}}$. For an isotropic system with extra spin rotation symmetry, the pseudo spin vector is $\boldsymbol{s_k} = \boldsymbol{s}_0 + \boldsymbol{s}_{\perp\alpha}\cos(W\theta_{\boldsymbol{k}}) + \boldsymbol{s}_{\perp\beta}\sin(W\theta_{\boldsymbol{k}})$, where $\boldsymbol{s}_{0,\perp\alpha,\perp\beta}$ are orthogonal each other,$|\boldsymbol{s}_0|$=$\sqrt{1-d^2_{\rm max}}$, and $|\boldsymbol{s}_{\perp\alpha}|$=$|\boldsymbol{s}_{\perp\beta}|$=$d_{\rm max}$. The momentum-dependent quantum distance is

$$d^2_{\boldsymbol{kk'}} = d^2_{\rm max}\frac{1-\cos W\theta_{kk'}}{2} \quad (29)$$

where where $d_{\max} = \max_{\boldsymbol{k}' \in \mathrm{FS}} [d_{\boldsymbol{k}\boldsymbol{k}'}]$ the maximum quantum distance on the Fermi surface, W is an integer.

$$\left\langle \frac{1}{\tau_k^{\mathrm{iso}}} \right\rangle = \sum_{k' \in \mathrm{FS}} \langle P_{kk'} \rangle (1 - \cos\theta_{kk'}), \tag{30}$$

$$= \frac{2\pi}{\hbar} \frac{\rho(\epsilon_F)}{2} \left[\gamma_0^2 (2 - d_{\max}^2) + \frac{|\gamma|^2}{3} (2 + d_{\max}^2) \right] + d_{\max}^2 \frac{2\pi}{\hbar} \rho(\epsilon_F) \frac{|\gamma|^2 - 3\gamma_0^2}{12} \delta_{W1}. \tag{31}$$

V. HIGHER ORDER CORRECTIONS TO THE FERMI GOLDEN RULE

The scattering probability involves the following quantity:

$$p_{kk'} = |\langle k'| (v_{q,i}\sigma_i) |k\rangle|^2 = \langle k'| (v_{q,i}\sigma_i) |k\rangle \langle k| (v_{-q,m}\sigma_m) |k'\rangle, \tag{32}$$

$$= \langle k'| (v_{q,i}\sigma_i) \frac{1}{2} (s_{k,j}\sigma_j) (v_{-q,m}\sigma_m) |k'\rangle, \tag{33}$$

$$= \frac{1}{2} (v_{q,i} v_{-q,m}) (s_{k,j} s_{k',l}), \tag{34}$$

where the summation over repeated indices ($i, j, m = \{0, x, y, z\}$) are omitted. $v_{q,0} = 1$ and $s_{k,0} = 1$. $s_{k',l} = \langle k'|\sigma_i \sigma_j \sigma_m|k'\rangle$. The scattering probability is expressed in terms of pseudospin vectors. After the disorder averaging, $\langle v_{q,i} v_{-q,m} \rangle = \gamma_i^2 \delta_{im}$. Then, if $j = i$, $s_{k',l} = s_{k',j}$, if $j \neq i$, $s_{k',l} = -s_{k',j}$. When $\gamma_x^2 = \gamma_y^2 = \gamma_z^2 = |\gamma|^2/3$, the above can be reduced to

$$p_{kk'} = \left[\frac{|\gamma|^2}{3} (1 + d_{\boldsymbol{k}\boldsymbol{k}'}^2) + \gamma_0^2 (1 - d_{\boldsymbol{k}\boldsymbol{k}'}^2) \right] \tag{35}$$

The next order is

$$p^{(2)}_{kk''k'} = |\langle k'| (v_{q,i}\sigma_i) |k''\rangle \langle k''| (v_{q',l}\sigma_l) |k\rangle|^2, \tag{36}$$

$$= \langle k'| (v_{q,i}\sigma_i) |k''\rangle \langle k''| (v_{q',l}\sigma_l) |k\rangle \langle k| (v_{-q',l'}\sigma_{l'}) |k''\rangle \langle k''| (v_{-q,i'}\sigma_{i'}) |k'\rangle, \tag{37}$$

$$= \langle k'| (v_{q,i}\sigma_i) \frac{1}{2} (s_{k'',j}\sigma_j) (v_{q',l}\sigma_l) \frac{1}{2} (s_{k,j'}\sigma_{j'}) (v_{-q',l'}\sigma_{l'}) \frac{1}{2} (s_{k'',j''}\sigma_{j''}) (v_{-q,i'}\sigma_{i'}) |k'\rangle, \tag{38}$$

$$= \frac{1}{8} (v_{q,i} v_{q',l} v_{-q',l'} v_{-q,i'}) (s_{k'',j} s_{k,j'} s_{k'',j''} s_{k',j'''}) \tag{39}$$

where $s_{k',j'''} = \langle k'|\sigma_i \sigma_j \sigma_l \sigma_{j'} \sigma_{l'} \sigma_{j''} \sigma_{i'}|k'\rangle$, $\boldsymbol{q} = \boldsymbol{k}' - \boldsymbol{k}''$, and $\boldsymbol{q}' = \boldsymbol{k}'' - \boldsymbol{k}$. They are all expressed in terms of the multiplication of pseudospin vectors. After the disorder averaging, the scattering probability becomes isotropic in spinor space, and then the scattering probability can be expressed in terms of the quantum distance $d^2_{kk''}$ and $d^2_{k'k''}$.

After taking the disorder averaging, we get

$$p^{(2)}_{kk''k'} = \langle v_{q,i} v_{q',l} v_{-q',l'} v_{-q,i'} \rangle \, \langle k'|\sigma_i|k''\rangle\langle k''|\sigma_l|k\rangle\langle k|\sigma_{l'}|k''\rangle\langle k''|\sigma_{i'}|k'\rangle, \tag{40}$$

$$\tag{41}$$

Here, $\langle v_{q,i} v_{q',l} v_{-q',l'} v_{-q,i'} \rangle = \Big(\langle v_{q,i} v_{q',l} \rangle \, \langle v_{-q',l'} v_{-q,i'} \rangle + \langle v_{q,i} v_{-q,i'} \rangle \, \langle v_{q',l} v_{-q',l'} \rangle + \langle v_{q,i} v_{-q',l'} \rangle \, \langle v_{q',l} v_{-q,i'} \rangle \Big)$, and we consider an approximation $\langle v_{q,i} v_{q',l} v_{-q',l'} v_{-q,i'} \rangle \approx \langle v_{q,i} v_{-q,i'} \rangle \, \langle v_{q',l} v_{-q',l'} \rangle$, since $\langle v_{q,i} v_{q',l} \rangle \propto \delta(\boldsymbol{k'} - \boldsymbol{k})$ and $\langle v_{q,i} v_{-q',l'} \rangle \propto \delta(\boldsymbol{k'} + \boldsymbol{k} - 2\boldsymbol{k''})$, and the related terms only give small deviations. Hence,

$$p^{(2)}_{kk''k'} = \langle v_{q,i} v_{-q,i'} \rangle \, \langle v_{q',l} v_{-q',l'} \rangle \, \langle k'|\sigma_i|k''\rangle\langle k''|\sigma_l|k\rangle\langle k|\sigma_{l'}|k''\rangle\langle k''|\sigma_{i'}|k'\rangle, \tag{42}$$

$$= \langle v_{q,i} v_{-q,i'} \rangle \, \langle k'|\sigma_i|k''\rangle\langle k''|\sigma_{i'}|k'\rangle \, \langle v_{q',l} v_{-q',l'} \rangle \, \langle k''|\sigma_l|k\rangle\langle k|\sigma_{l'}|k''\rangle, \tag{43}$$

$$= \Big[\frac{|\gamma|^2}{3}(1 + d^2_{\boldsymbol{k'k''}}) + \gamma_0^2(1 - d^2_{\boldsymbol{k'k''}}) \Big] \Big[\frac{|\gamma|^2}{3}(1 + d^2_{\boldsymbol{kk''}}) + \gamma_0^2(1 - d^2_{\boldsymbol{kk''}}) \Big]. \tag{44}$$

That is, $p^{(2)}_{kk''k'} \simeq p_{kk''} p_{k''k'}$.

The Fermi Golden rule, including the higher order corrections[3], is

$$P_{kk'} = \frac{2\pi}{\hbar} |\langle k'| U_{\text{pert}} \left(I - G_0^{\text{ret}} U_{\text{pert}} \right)^{-1} |k\rangle|^2 \delta(\epsilon_k - \epsilon_{k'}), \tag{45}$$

$$= \frac{2\pi}{\hbar} \delta(\epsilon_k - \epsilon_{k'}) \sum_{n=1,2,\cdots} P^{(n)}_{kk'}, \tag{46}$$

where $G_0^{\text{ret}} = \sum_{k''} |k''\rangle\langle k''|/(\epsilon_k - \epsilon_{k''} + i0^+)$, and

$$P^{(1)}_{kk'} = p_{kk'}, \tag{47}$$

$$P^{(2)}_{kk'} = \sum_{k''} \frac{w^{(2)}_{kk''k'}}{(\epsilon_k - \epsilon_{k''} + i0^+)(\epsilon_k - \epsilon_{k''} - i0^+)}. \tag{48}$$

After disorder averaging, the transport scattering rate $\langle 1/\tau_k \rangle$ will be in terms of $\sum_{k' \in \text{FS}} d^2_{kk'}$, $\sum_{k',k'' \in \text{FS}} d^2_{kk''} d^2_{k''k'}$, etc. As a result, the distribution of quantum distance on the Fermi surface (including higher moments) will be reflected in the rate.

Dressed Green function: Consider the following quantity.

$$\langle k'|T_+|k\rangle\langle k|T_-|k'\rangle = \langle k'| U_{\text{pert}} \left(I - G_0^r U_{\text{pert}} \right)^{-1} |k\rangle\langle k| U_{\text{pert}} \left(I - G_0^a U_{\text{pert}} \right)^{-1} |k'\rangle. \tag{49}$$

When one performs the disorder averaging, if the pairing of two scattering potentials is selected in the first (second) T-matrix T_+ (T_-), it renormalizes the retarded (advanced) Green function:

$$G^r = (\epsilon - H_0 - \Sigma^r)^{-1}, \tag{50}$$

$$= G_0^r + G_0^r \Sigma^r G_0^r + G_0^r \Sigma^r G_0^r \Sigma^r G_0^r + \cdots, \tag{51}$$

where $G_0^r = \sum_k \frac{|k\rangle\langle k|}{\epsilon-\epsilon_k+i0^+}$, where the self-energy can be obtained by collecting non-crossing diagrams

$$\Sigma^r = \sum_{i=0,x,y,z} (v_{q,i}\sigma_i)G_0^r(v_{-q,i}\sigma_i), \tag{52}$$

$$= \sum_{i=0,x,y,z} |v_{q,i}|^2 \sum_k \frac{\sigma_i|k\rangle\langle k|\sigma_i}{\epsilon-\epsilon_k+i0^+}, \tag{53}$$

$$= \sum_{i,j=0,x,y,z} |v_{q,i}|^2 \sum_k \frac{\sigma_i\frac{1}{2}s_{k,j}\sigma_j\sigma_i}{\epsilon-\epsilon_k+i0^+}, \tag{54}$$

where $|k\rangle\langle k| = \sum_{j=0,x,y,z}\frac{1}{2}s_{k,j}\sigma_j$ and $s_{k,0} = 1$. The summation over momentum k can be split into the integration over angle θ_k and the magnitude $|k|$. Then, the latter is converted to energy integration. We focus on the imaginary part of the self-energy:

$$\text{Im}[\Sigma^r] = -\pi\rho(\epsilon_F)\sum_{i,j=0,x,y,z}\sigma_i\sigma_j\sigma_i|v_{q,i}|^2\int\frac{d\theta_k}{2\pi}\frac{1}{2}s_{\theta_k,j}, \tag{55}$$

which contains a typical pseudospin independent component ($\sim \sigma_0$) and dependent component ($\sim \sigma_{\hat{n}}$), where $\hat{n}$ is the averaged pseudospin direction on the Fermi surface. Explicitly,

$$\text{Im}[\Sigma^r] = -\frac{\pi}{2}\rho(\epsilon_F)\left[\sum_{i=0,x,y,z}|v_{q,i}|^2\sigma_0 + \sum_{j=x,y,z}\left(|v_{q,0}|^2 - |\boldsymbol{v}_q|^2 + 2|v_{q,j}|^2\right)\sigma_j\int\frac{d\theta_k}{2\pi}s_{\theta_k,j}\right], \tag{56}$$

$$= -\frac{\pi}{2}\rho(\epsilon_F)\left[\gamma_0^2(\sigma_0 + \sum_{j=x,y,z}\sigma_j\int\frac{d\theta_k}{2\pi}s_{\theta_k,j}) + \frac{1}{3}|\boldsymbol{\gamma}|^2\sum_{j=x,y,z}(\sigma_0 - \sigma_j\int\frac{d\theta_k}{2\pi}s_{\theta_k,j})\right] \tag{57}$$

where in the second line $|v_{q,0}|^2{=}\gamma_0^2$ and $|v_{q,x}|^2{=}|v_{q,y}|^2{=}|v_{q,z}|^2{=}\frac{1}{3}|\boldsymbol{\gamma}|^2$ are used. The pseudospin-dependent scattering rate is so interesting that we leave it for future study. In the following, we consider a situation with $\int d\theta_k s_{\theta_k,j} = 0$ and therefore $\text{Im}[\Sigma^r] = -\frac{\pi}{2}\rho(\epsilon_F)(\gamma_0^2 + |\boldsymbol{\gamma}|^2)\sigma_0$. This is the case when pseudospin $\boldsymbol{s}$ makes a circle with $\boldsymbol{s}_0 = 0$. In this case, even though $d_{\text{max}} = 1$, we symbolically keep the notation. The computation for $d_{\text{max}} < 1$ will require more careful treatment of the pseudospin structure of Σ^r.

The vertex correction: Let us consider the pairing of two scattering potentials where one is selected

from T_+ and the other from T_-. Using the disorder averaged Green's function $G^{r,a}$,

$$P^{(2)}_{kk'} = \sum_{k''} \frac{w^{(2)}_{kk''k'}}{(\epsilon_k - \epsilon_{k''} + i\eta)(\epsilon_k - \epsilon_{k''} - i\eta)}, \tag{58}$$

$$= \int \frac{d\theta_{k''}}{2\pi} w^{(2)}_{\theta_k\theta_{k''}\theta k'} \int d\epsilon_{k''}\rho(\epsilon_{k''}) \frac{1}{(\epsilon_k - \epsilon_{k''} + i\eta)(\epsilon_k - \epsilon_{k''} - i\eta)}, \tag{59}$$

$$= \int \frac{d\theta_{k''}}{2\pi} w^{(2)}_{\theta_k\theta_{k''}\theta k'} \int d\epsilon_{k''}\rho(\epsilon_{k''}) \left[\frac{1}{\epsilon_k - \epsilon_{k''} - i\eta} - \frac{1}{\epsilon_k - \epsilon_{k''} + i\eta}\right] \frac{1}{2i\eta}, \tag{60}$$

$$= \frac{2}{\gamma_0^2 + |\boldsymbol{\gamma}|^2} \int \frac{d\theta_{k''}}{2\pi} w^{(2)}_{\theta_k\theta_{k''}\theta_{k'}}, \tag{61}$$

where in the second line we consider $p^{(2)}_{kk''k'} = p^{(2)}_{\theta_k\theta_{k''}\theta k'}$ is only a function of angles of momentum. In the third line, $\eta = -\text{Tr Im}\Sigma^r = \pi\rho(\epsilon_F)(\gamma_0^2 + |\boldsymbol{\gamma}|^2)$. The above calculation indicates that $P^{(1)}_{kk'}$ and $P^{(2)}_{kk'}$ share the same scaling on the disorder strength, $\sim \gamma_j^2$.

$$P^{(2)}_{kk'} \simeq \frac{1}{\gamma_0^2 + |\boldsymbol{\gamma}|^2} \int \frac{d\theta_{k''}}{2\pi} p_{kk''} p_{k''k'}, \tag{62}$$

One can continue to take into account higher-order processes.

$$P^{(3)}_{kk'} \simeq \left(\frac{1}{\gamma_0^2 + |\boldsymbol{\gamma}|^2}\right)^2 \int \frac{d\theta_{k_2}}{2\pi} \int \frac{d\theta_{k_3}}{2\pi} p_{kk_2} p_{k_2k_3} p_{k_3k}, \tag{63}$$

$$P^{(n)}_{kk'} \simeq \left(\frac{1}{\gamma_0^2 + |\boldsymbol{\gamma}|^2}\right)^{n-1} \int \prod_{j=2}^{n} \left(\frac{d\theta_{k_j}}{2\pi}\right) p_{kk_2} p_{k_2k_3} \cdots p_{k_jk_{j+1}} \cdots p_{k_nk}, \tag{64}$$

which include $2n$ power of quantum distances. The disorder averaged generalized Fermi Golden rule is the summation of them all (46). As a result, including all powers of disorder scatterings, the distribution of quantum distances on the Fermi surface determines the transition probability. That is, the information of whole moments of quantum distances is involved in the transition probability

Example: Isotropic model:– When a system has the orbital-spin rotation symmetry by $\hat{U}_{\theta_k,W}$, the higher order multiplication of quantum distances can be lowered down to the quadratic:

$$\int \frac{d\theta_{k''}}{2\pi} \left(1 - 2\frac{d^2_{kk''}}{d^2_{\text{max}}}\right)\left(1 - 2\frac{d^2_{k''k'}}{d^2_{\text{max}}}\right) = \int \frac{d\theta_{k''}}{2\pi} \cos W\theta_{kk''} \cos W\theta_{k''k'} = \frac{1}{2}\left(1 - 2\frac{d^2_{kk'}}{d^2_{\text{max}}}\right) \tag{65}$$

Try to reduce the following:

$$p_{kk'} = \gamma_s^2 \frac{2+d_{\max}^2}{2} + \gamma_0^2 \frac{2-d_{\max}^2}{2} + \frac{\gamma_0^2-\gamma_s^2}{2} d_{\max}^2 \cos W\theta_{kk'}, \tag{66}$$

$$\int \frac{d\theta_{k_2}}{2\pi} p_{kk_2} p_{k_2k'} = \left[\gamma_s^2 \frac{2+d_{\max}^2}{2} + \gamma_0^2 \frac{2-d_{\max}^2}{2}\right]^2 + \left[\frac{\gamma_0^2-\gamma_s^2}{2} d_{\max}^2\right]^2 \frac{1}{2} \cos W\theta_{kk'}, \tag{67}$$

$$\int \prod_{j=2}^{n} \left(\frac{d\theta_{k_j}}{2\pi}\right) p_{kk_2} \cdots\cdots p_{k_nk} = \left[\gamma_s^2 \frac{2+d_{\max}^2}{2} + \gamma_0^2 \frac{2-d_{\max}^2}{2}\right]^n + \left[\frac{\gamma_0^2-\gamma_s^2}{2} d_{\max}^2\right]^n \frac{1}{2^{n-1}} \cos W\theta_{kk'}, \tag{68}$$

where $\gamma_s^2 = |\boldsymbol{\gamma}|^2/3$ is introduced. As a result,

$$P_{kk'} = \frac{2\pi}{\hbar} \delta(\epsilon_k - \epsilon_{k'}) \sum_{n=1,2,\cdots} P_{kk'}^{(n)}, \tag{69}$$

$$= \frac{2\pi}{\hbar} \delta(\epsilon_k - \epsilon_{k'}) \sum_{n=1,2,\cdots} \left(\frac{1}{\gamma_0^2 + 3\gamma_s^2}\right)^{n-1} \left\{ \left[\gamma_s^2 \frac{2+d_{\max}^2}{2} + \gamma_0^2 \frac{2-d_{\max}^2}{2}\right]^n + \left[\frac{\gamma_0^2-\gamma_s^2}{2} d_{\max}^2\right]^n \frac{1}{2^{n-1}} \cos W\theta_{kk'} \right\}, \tag{70}$$

which is the scattering probability, including all orders of disorder scatterings (taking non-crossing diagrams). We can next obtain the transport scattering from Eq. (19) in the main text. The summation can be performed and note that we assumed $d_{\max} = 1$ which makes $\mathrm{Im}[\Sigma^r] = -\frac{\pi}{2}\rho(\epsilon_F)(\gamma_0^2 + |\boldsymbol{\gamma}|^2)\sigma_0$:

$$\frac{1}{\tau_k^{(sc)}} = \sum_{k'} P_{kk'}, \tag{71}$$

$$= \frac{2\pi}{\hbar}\rho(\epsilon_F)\left(\gamma_s^2 \frac{2+d_{\max}^2}{2} + \gamma_0^2 \frac{2-d_{\max}^2}{2}\right)\left[1 - \frac{1}{\gamma_0^2 + 3\gamma_s^2}(\gamma_s^2 \frac{2+d_{\max}^2}{2} + \gamma_0^2 \frac{2-d_{\max}^2}{2})\right]^{-1}, \tag{72}$$

$$\frac{1}{\tau_k^{(tr)}} = \frac{1}{\tau_k^{(sc)}} - \frac{2\pi}{\hbar}\rho(\epsilon_F)\frac{d_{\max}^2(\gamma_0^2-\gamma_s^2)}{4 + d_{\max}^2(\gamma_0^2-\gamma_s^2)}\delta_{W1}, \tag{73}$$

VI. COULOMB-TYPE IMPURITY POTENTIAL IN ISOTROPIC QUADRATIC BAND-TOUCHING MODEL

In the case of a Coulomb-type impurity potential , $\gamma_0 = v_{\boldsymbol{q},0}$ is a function of momentum $\boldsymbol{q}$. The Fourier transformed potential becomes $v_{\boldsymbol{q},0} = 2\pi e^2/\epsilon_0(|\boldsymbol{q}| + q_s)\sigma_0$, and $P_{kk'}$ becomes as follows:

$$P_{\boldsymbol{k'k}} = n_{\text{imp}} \frac{2\pi}{\hbar} \tilde{v}_{\text{imp}}(\boldsymbol{k} - \boldsymbol{k'})^2 \left[1 - d_{\text{HS}}^2(\boldsymbol{k}, \boldsymbol{k'})\right] \delta(\epsilon_{n,\boldsymbol{k'}} - \epsilon_{n,\boldsymbol{k}}), \tag{74}$$

where $n_{\rm imp}$ is the density of impurities. The static dielectric function is written as

$$\epsilon(\boldsymbol{q}) = 1 + \frac{2\pi e^2}{\epsilon_0 q}\Pi(q), \tag{75}$$

where $\Pi(q)$ is the polarization function. In the long-wavelength limit, the dielectric function is written as

$$\epsilon(q) = 1 + \frac{2\pi e^2}{\epsilon_0 q}\Pi(0), \tag{76}$$

The screening constant q_s is written as

$$q_s = \frac{2\pi e^2}{\epsilon_0}\Pi(0). \tag{77}$$

From the compressibility sum-rule,

$$\Pi(0) = \int_{-\infty}^{\infty}(-\frac{\partial f^{(0)}(\epsilon)}{\partial \epsilon})\rho(\epsilon)d\epsilon, \tag{78}$$

where $\rho(\epsilon)$ is the density of states. Therefore, $q_s = 2\pi e^2\rho(\epsilon_F)/\epsilon_0 = 2me^2/\epsilon$, where m is the effective mass.

If we consider the quadratic isotropic band-touching model, we get

$$\frac{1}{\tau_{\boldsymbol{k}_F}} = n_{\rm imp}\frac{2\pi}{\hbar}\frac{e^4}{\epsilon_0^2}\frac{2-d_{\rm max}^2}{16\pi k^2} \quad {\rm for} k_F \gg q_s, \tag{79}$$

and

$$\frac{1}{\tau_{\boldsymbol{k}_F}} = n_{\rm imp}\frac{2\pi}{\hbar}\frac{e^4}{\epsilon_0^2}\frac{2-d_{\rm max}^2}{8\pi q_s^2} \quad {\rm for} k_F \ll q_s. \tag{80}$$

We consider the limit of $k_F \gg q_s$, where there is no screening effect. (The case of $k_F \ll q_s$ is in the main text.) We calculate the response functions L_{ij} with eq. (79) changing the chemical potential μ and $d_{\rm max}$, as shown in Supporting Fig. 1. If we consider the ratio of $PF(d_{\rm max})$ and $PF(d_{\rm max} = 0)$, one get

$$PF(d_{\rm max}) = \frac{2}{2-d_{\rm max}^2}PF(d_{\rm max} = 0). \tag{81}$$

This is the same result in the case of $k_F \ll q_s$.

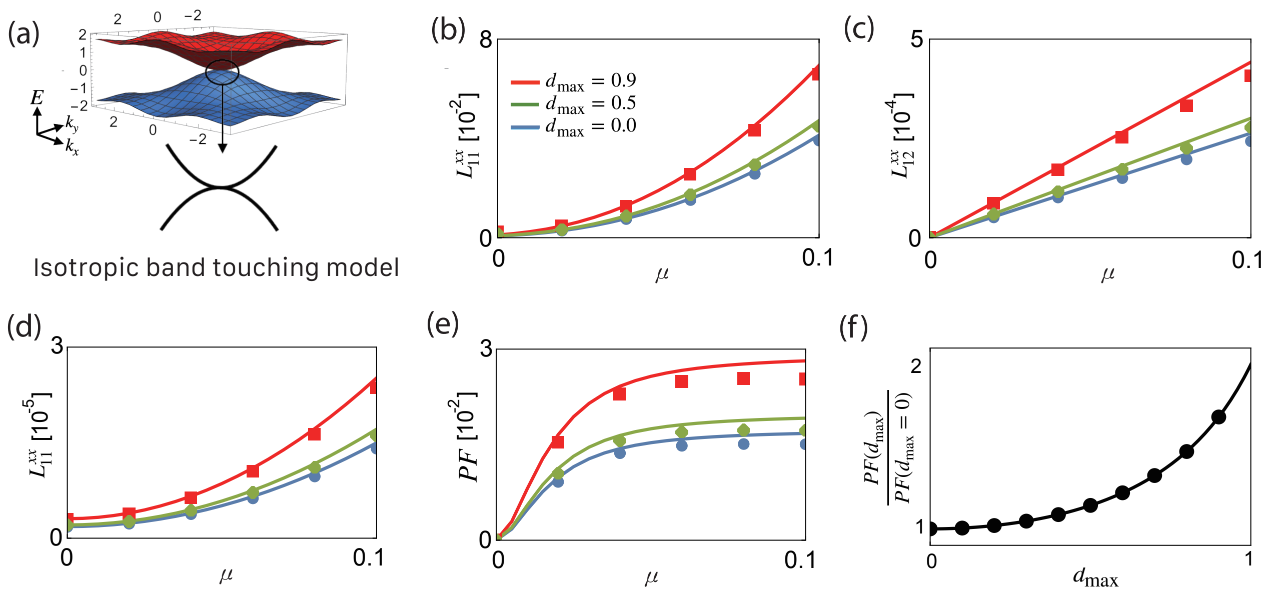

Supporting Figure 1: (a) Band structure of eq. (22) with $d_{\max} = 0.9$ in the main text. (b-e) Chemical potential μ dependence of (b) L_{11}, (c) L_{12}, (d) L_{22} and (e) PF for $d_{\max} = 0$(blue), 0.5(green) and 1(red). (f) represents the ratio between $PF(d_{\max})$ and $PF(0)$. The solid lines represent the results from the isotropic quadratic touching model in Eq. (18) in the main text with $m_+^{-1} = -m_-^{-1} = 1$ The discrete plots represent the results that are calculated from the lattice model Eq. (22) in the main text. In this calculation, we set the parameters $T = 0.01$, $k_B = \hbar = \gamma = u_0 = e = \epsilon = 1$, and consider the Coulomb-type impurity potential with $q_s \ll k_F$.

VII. TEMPERATURE DEPENDENCE OF TRANSPORT PROPERTIES

In the analysis of thermoelectric performance, the temperature is not only involved as ∇T across a device, but the Fermi-Dirac distribution of electrons and the strength of phonon-phonon interaction are temperature dependent as well. In this section, we compute Onsager coefficients and power factor for the isotropic quadratic band-touching model employed in Eq. (18) of the main text as illustrated in Supporting Fig. 2. We set $k_B = \hbar = e = 1$ unit, and the dimensionless value temperature $T = 0.01$ is roughly 116 (Kelvin).

Supporting Figures 2(a), (b), and (c) highlight the behavior of the thermal conductivity coefficient L_{11}, L_{12}, and L_{22} as a function of the chemical potential μ for $d_{\max} = 0, 0.5$ and 1, respectively. Supporting Figures 2(e), (f), and (g) show the $d_{\max}$-dependence of the thermal conductivity coefficient L_{11}, L_{12}, and L_{22}, respectively. As temperature increases, these coefficients

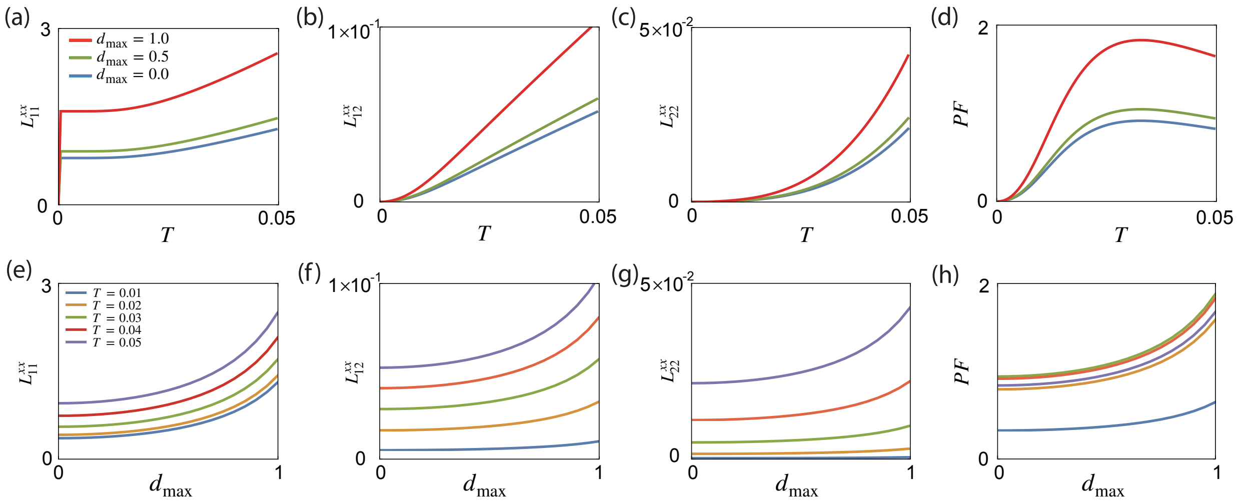

Supporting Figure 2: Temperature T dependence of Onsager coefficients and power factor (a) L_{11}. (b) L_{12}, (c) L_{22}, and (d) PF for $d_{\rm max} = 0$ (blue), 0.5 (green), and 1 (red). $d_{\rm max}$ dependence of (e) L_{11}, (f) L_{12}, (g) L_{22} and (h) PF for $T = 0.01$ (blue), 0.02 (orange), 0.03 (green), 0.04 (red) and 0.05 (purple). Here, we set $\mu = 0.05$, $k_B = \hbar = e = 1$, $1/\tau(d_{\rm max} = 0) = 0.01$, $m_+ = -m_- = 1$ for all calculations. The calculations are conducted with the isotropic quadratic touching model in Eq. (18).

also increase, and similarly, higher values of $d_{\rm max}$ lead to enhanced transport coefficients. Similar to μ dependence of PF, T dependence of PF has a maximum at a certain temperature, as shown in Supporting Figs. 2(d) and (h). This can be understood by T dependences of L_{11} and L_{12}. At low T, $L_{11} \propto T^0$, while $L_{12} \propto T^n (n \geq 2)$. On the other hand, at high T, $L_{11} \propto T$ and $L_{12} \propto T$. Since PF is expressed as $PF = (L_{12})^2/(L_{11}T^2)$, at low T, $PF \propto T^{(2n-2)}$, while $PF \propto T^{-1}$. This explains why PF has a maximum at a specific temperature. These results indicate that in addition to quantum geometric properties, temperature must be carefully considered when optimizing the performance of thermoelectric materials.

VIII. FIGURE OF MERIT (ZT) AND THERMAL CONDUCTIVITY FROM PHONON

The thermal conductivity κ consists of contributions from various factors, such as phonons and electrons. In typical metallic systems, thermal conductivity is primarily dominated by phonons and electrons; therefore, we consider the case where $\kappa = \kappa_{ph} + \kappa_e$, with κ_{ph} and κ_e representing the thermal conductivities due to phonons and electrons, respectively. Since κ_e is influenced by the electron scattering time, changes in the geometry of electronic wavefunctions can also alter κ_e, as shown in Supporting Figs. 3(a) and (b). In contrast, κ_{ph} is independent of the geometry of electronic wavefunctions. However, κ_{ph} significantly impacts ZT, as illustrated in Supporting Figs. 3(b) and (d).

The results indicate that the enhancement from controlling the geometry will have a greater impact when κ_{ph} is much larger than κ_e. In general, phonons typically dominate heat transport in semiconductors and semimetals[4–7]. In impure metals or disordered alloys, the electron mean free path is reduced due to collisions with impurities, making the phonon contribution comparable to the electronic contribution[8]. In pure metals with weak phonon-phonon scattering, κ_{ph} can be comparable to, or even exceed, the electronic contribution[9,10]. In such materials, an increase in PF would significantly enhance ZT.

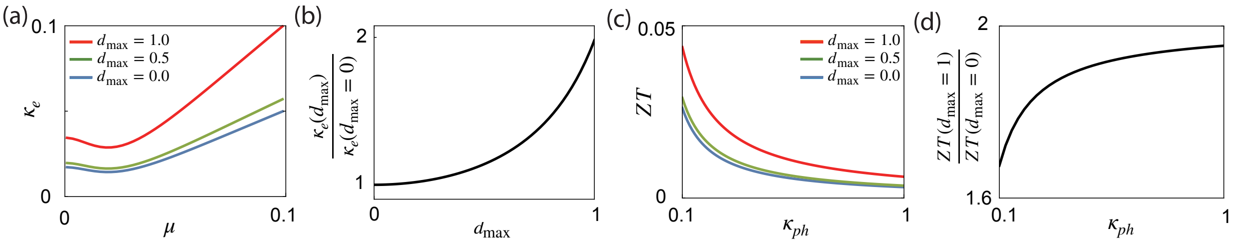

Supporting Figure 3: (a) μ-dependence of κ_e for $d_{\max} = 0$ (blue), 0.5(green) and 1 (red), respectively. (b) $d_{\max}$-dependence of $\kappa_e/\kappa_e(d_{\max} = 0)$. (c) κ_{ph}-dependence of ZT for $d_{\max} = 0$ (blue), 0.5(green) and 1 (red), respectively. (d) The ratio of ZT between $d_{\max} = 0$ and 1 as a function of κ_{ph}. Here, we set $T = 0.01$, $k_B = \hbar = e = 1$, $1/\tau(d_{\max} = 0) = 0.01$, $m_+ = -m_- = 1$ for all calculations, and $\mu = 0.05$ for (b),(c) and (d). The calculations are conducted with the isotropic quadratic touching model in Eq. (18) in the main text.

IX. EXAMPLE: A 3-DIMENSIONAL ISOTROPIC BAND-TOUCHING MODEL

The concept of quantum distance and its influence on the transport scattering rate is not limited to the 2D example discussed in the main manuscript. In fact, our discussion up to Eq.(13) of the main text holds for a two-band model system in a higher dimension as well. Below, we provide 3-dimensional examples where the role of quantum distance can be specifically observed from quantum transport experiments. Note that the 3-dimensional isotropic band-touching model with nonzero quantum distance is neither theoretically nor experimentally discussed in the literature. Consider the following Hamiltonians:

$$H_0 = -(k_x^2 + k_y^2)/4\,\sigma_0 + k_z^2/2\,\sigma_1 - (k_x^2 + k_y^2)/4\,\sigma_3, \tag{82}$$

$$H_1 = -(k_x^2 + k_y^2)/4\,\sigma_0 + k_z^2/2\,\sigma_1 - k_x k_y/2\,\sigma_2 - (k_x^2 - k_y^2)/4\,\sigma_3. \tag{83}$$

When $k_z = 0$, H_0 and H_1 correspond to $d_{\text{max}} = 0$ and $d_{\text{max}} = 1$ for the Hamiltonian given in Eq. (18) with $m_+ = -m_- = 1$.As a result, the eigenenergies for both models are identical:

$$E^{\pm}(k_x, k_y, k_Z) = -\frac{k_x^2 + k_y^2}{4} \pm \sqrt{(k_x^2 + k_y^2)^2 + 4k_z}. \tag{84}$$

However, the two models exhibit entirely different (pseudo-)spin textures and, therefore, transport properties, as illustrated in Supporting Fig. 4. This indicates that nontrivial quantum geometry can alter thermoelectric performance in 3-dimensional systems.

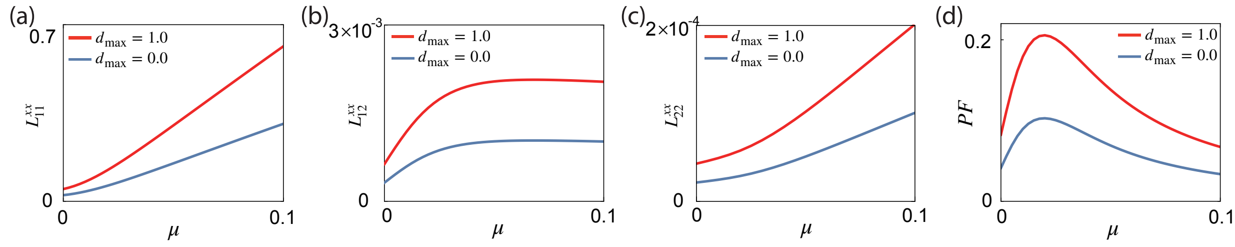

Supporting Figure 4: Onsager coefficients and power factor (a) L_{11}, (b) L_{12}, (c) L_{22} and (d) PF as a function of chemical potential μ. The blue and red lines represent the results of H_0 ($d_{\text{max}} = 0$) and H_1 ($d_{\text{max}} = 1$), respectively. $T = 0.01$, $k_B = \hbar = e = 1$, and $1/\tau(d_{\text{max}} = 0) = 0.01$ are used.

X. TRANSPORT PROPERTIES IN BILAYER GRAPHENE

As an application to a real material, we consider Bernal-stacked bilayer graphene. For $d_{\text{max}} = 1$ and $m_+ = -m_- = \gamma/(2\hbar^2 v_F^2)$, the model in Eq. (18) of the main text represents the effective

Hamiltonian of the bilayer graphene[11]. In the Reference[12], the conductivity and Seebeck coefficient as a function of the back-gate voltage (V_{BG}) at various temperatures are measured experimentally. The phonon-drag effect was not observed, indicating weak electron-phonon coupling in the bilayer graphene. This further supports and demonstrates the validity of the Boltzmann theory in this system. In this section, we demonstrate that the transport properties of the bilayer graphene are strongly correlated with its geometric characteristics.

We compare the cases for $d_{\max} = 0$ and $d_{\max} = 1$, fixing the parameters as $v_F = 0.95 \times 10^6$ and $\gamma = 0.39\text{eV}$, which are consistent values used in[12]. Since the Boltzmann theory excellently explains the experimental results at low temperatures (below 140 K), we focus on a temperature of 70 K. Supporting Figures. 5 (a), (b), and (c) show the resistivity, Seebeck coefficient, and PF for $d_{\max} = 0$ and 1, respectively. The case of $d_{\max} = 1$ is consistent with previous studies[12,13], particularly at low V_{BG}. For $d_{\max} = 0$, the conductivity and PF are half of those for $d_{\max} = 1$. This indicates the geometry of wavefunctions plays a significant role in the bilayer graphene's transport properties. However, the Seebeck coefficient remains the same for both $d_{\max} = 0$ and $d_{\max} = 1$. This is because S is defined as $S = L_{12}/(L_{11}T)$, meaning the geometric effect in $1/\tau$ cancels out, leaving S unaffected by the quantum geometry.

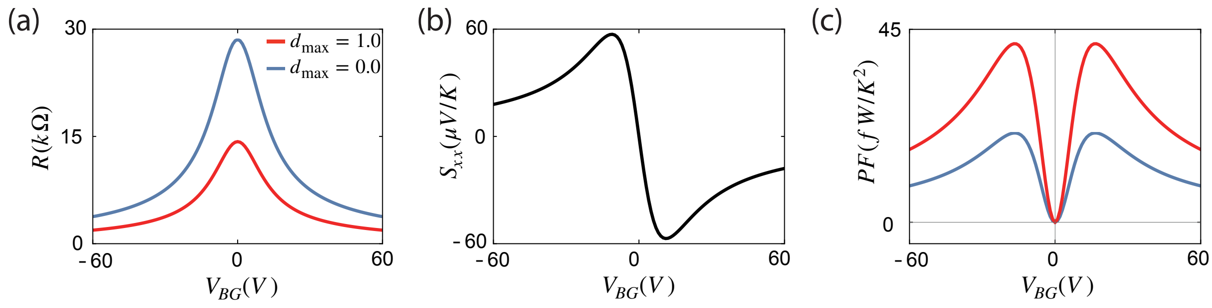

Supporting Figure 5: (a) Resistance R, (b) Seebeck coefficient S and (c) Power factor PF as a function of backgate voltage V_{BG} at temperature of 70 K. The red and blue lines in (a) and (c) represent the cases of $d_{\max} = 1$ and $d_{\max} = 0$, respectively. For (b), both cases of $d_{\max} = 1$ and $d_{\max} = 0$ exhibit the same results.

XI. TRANSPORT SCATTERING RATE FOR A SINGLE SCATTERING STRENGTH

Well-known types of scattering sources include impurities, defects of a crystalline, vacancies, adatoms, and lattice mismatches at interfaces. Such scatterers are present with different amounts of materials grown in different ways. Though it is not possible to build a general theory taking into account all those conditions, as a first approximation, we consider the randomness of scatterers to be distributed according to the Gaussian function in the main manuscript. This can take into account the average and the variance (the first and the second cumulant of a distribution) of the disorder ensemble. In this section, we compute the transport scattering probability for either nonmagnetic or magnetic scattering potential with a single strength instead of a Gaussian distribution. This situation can be viewed as a disorder distribution with a high skewness. The expression Eq.(12) of the main text is valid in general for any two-band systems. When specific information of disorder distribution is available, one can use it to produce the transport scattering rate and its dependence on the quantum distance.

For a nonmagnetic scattering potential ($v_{\boldsymbol{q},i=x,y,z} = \mathbf{0}$), the scattering probability simplifies to

$$P_{\boldsymbol{k}'\boldsymbol{k}} = -\frac{2\pi}{\hbar}\left[|v_{\boldsymbol{q},0}|^2\frac{1-\boldsymbol{s}_{\boldsymbol{k}}\cdot\boldsymbol{s}_{\boldsymbol{k}'}}{2}\right]\delta(\epsilon_{\boldsymbol{k}'}-\epsilon_{\boldsymbol{k}}). \tag{85}$$

where $|v_{\boldsymbol{q},0}|^2$ is a nonmagnetic scattering strength. A straightforward calculation for an isotropic 2D two-band model leads to

$$1/\tau_{\boldsymbol{k}} = 2\pi^2|v_{\boldsymbol{q},0}|^2 n_{imp}(2-d_{\max}^2). \tag{86}$$

If we set $1/\tau_{\boldsymbol{k}}(d_{\max}=0) = 0.01$, the response functions correspond to the results shown in Fig. 3 of the main text.

For a magnetic scattering potential, where $v_{\boldsymbol{q},0} = 0$, $v_{\boldsymbol{q},x} = V\sin\theta\cos\phi$, $v_{\boldsymbol{q},y} = V\sin\theta\sin\phi$ and $v_{\boldsymbol{q},z} = V\cos\theta$, the scattering probability becomes

$$P_{\boldsymbol{k}'\boldsymbol{k}} = \frac{2\pi}{\hbar}\left[V^2\frac{1-\boldsymbol{s}_{\boldsymbol{k}}\cdot\boldsymbol{s}_{\boldsymbol{k}'}}{2} + \sum_{i,j=x,y,z}(v_{\boldsymbol{q},i}\boldsymbol{s}_{\boldsymbol{k},i})(v_{\boldsymbol{q},j}\boldsymbol{s}_{\boldsymbol{k}',j})\right]\delta(\epsilon_{\boldsymbol{k}'}-\epsilon_{\boldsymbol{k}}). \tag{87}$$

where a short range ($\boldsymbol{q}$-independent) scattering potential is assumed (See Supporting Information

Sec. VI for Coulomb-type long-range scattering potential). A straightforward calculation yields

$$\begin{aligned}1/\tau_{\boldsymbol{k}} =& n_{\text{imp}}\pi^2V^2\Big[2+2\cos(2\theta)-5d_{\max}^2\cos(2\theta)+3d_{\max}^2\cos(2\theta)\cos(2\phi_{\boldsymbol{k}})\\ &+6d_{\max}^4\cos(2\theta)\sin^2(2\phi_{\boldsymbol{k}})+4d_{\max}\sqrt{1-d_{\max}^2}\cos\phi\sin\theta\{(2-2d_{\max}^2)\cos\theta\\ &+(2d_{\max}^2-1)\cos\theta\cos\phi_{\boldsymbol{k}}+d_{\max}\sin\theta\sin\phi\sin(2\phi_{\boldsymbol{k}})\}\\ &+2d_{\max}(1-d_{\max})\{d_{\max}\left(-1+2\cos(2\phi)\sin^2\theta\right)\sin^2\phi_{\boldsymbol{k}}+\sin(2\theta)\sin\phi\sin(2\phi_{\boldsymbol{k}})\}\Big],\end{aligned} \tag{88}$$

where $\phi_{\boldsymbol{k}}$ is defined from $\boldsymbol{k}=(k\cos\phi_{\boldsymbol{k}}, k\sin\phi_{\boldsymbol{k}})$. For instance, for magnetic impurities aligned along $x-$direction ($\theta=\pi/2, \phi=0$), $y-$direction ($\theta=\pi/2, \phi=\pi/2$) and $z-$direction ($\theta=0$), the scattering rates are, respectively,

$$1/\tau_{\boldsymbol{k}} = 2n_{\text{imp}}\pi^2V^2d_{\max}^2[3-2d_{\max}^2+2(-1+d_{\max}^2)\cos 2\phi_{\boldsymbol{k}}] \quad (x \text{ direction}) \tag{89}$$

$$1/\tau_{\boldsymbol{k}} = 2n_{\text{imp}}\pi^2V^2d_{\max}^2 \quad (y \text{ direction}) \tag{90}$$

$$1/\tau_{\boldsymbol{k}} = -2n_{\text{imp}}\pi^2V^2[-2+3d_{\max}^2-2d_{\max}^4+2d_{\max}^2(-1+d_{\max}^2)\cos 2\phi_{\boldsymbol{k}}] \quad (z \text{ direction}). \tag{91}$$

With these scattering rates, we calculate $d_{\max}$ dependence of PF. Supporting Figure 6 shows that the scattering probability and, therefore, the power factor is sensitively dependent on the direction of the scatter's magnetism. Additionally, when $d_{\max}=0$, the spins are all aligned in the z-direction. As a result, magnetic impurities in the x and y directions do not induce scattering since the spin orientation is orthogonal to the impurity field. This explains why the power factor diverges as $d_{\max}$ approaches zero in Supporting Figs. 6 (a) and (b).

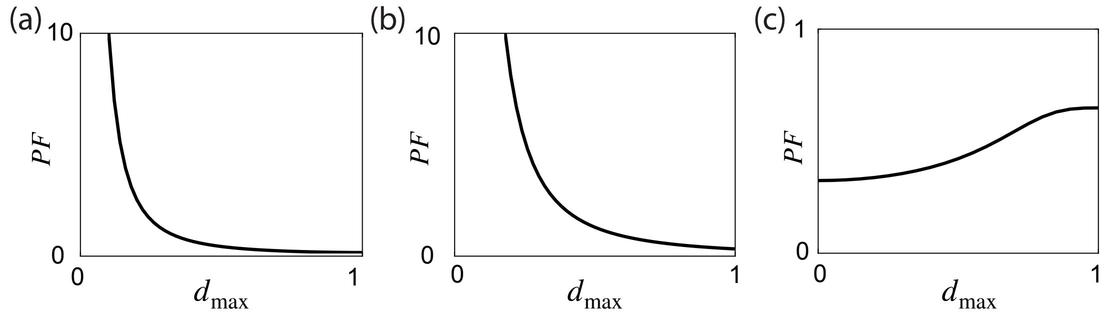

Supporting Figure 6: Quantum distance $d_{\max}$-dependence of the power factor PF for (a) x-direction, (b) y-direction, and (c) z-direction magnetic impurities, respectively. $T=0.01$, $k_B=\hbar=e=1$, $\mu=0.05$, and $1/\tau(d_{\max}=1)=0.02$ are used.

[1] J.-W. Rhim and B.-J. Yang, Physical Review B **99**, 045107 (2019).

[2] L. Onsager, Physical review **37**, 405 (1931).

[3] H.-Y. Wang, Journal of Physics Communications **7**, 075001 (2023).

[4] M. Holland, Physical Review **134**, A471 (1964).

[5] L. Lindsay and D. Broido, Journal of Physics: Condensed Matter **20**, 165209 (2008).

[6] Y. Hu, X. Ding, X. Jin, R. Wang, X. Yang, and X. Zhou, Applied Physics Letters **125** (2024).

[7] S. Yue, H. T. Chorsi, M. Goyal, T. Schumann, R. Yang, T. Xu, B. Deng, S. Stemmer, J. A. Schuller, and B. Liao, Physical Review Research **1**, 033101 (2019).

[8] C. Kittel, *Introduction to solid state physics* (John Wiley & sons, inc, 2005).

[9] Y. Chen, J. Ma, and W. Li, Physical Review B **99**, 020305 (2019).

[10] A. Kundu, J. Ma, J. Carrete, G. Madsen, and W. Li, Materials Today Physics **13**, 100214 (2020).

[11] E. McCann and M. Koshino, Reports on Progress in physics **76**, 056503 (2013).

[12] S.-G. Nam, D.-K. Ki, and H.-J. Lee, Phys. Rev. B **82**, 245416 (2010).

[13] S. Morozov, K. Novoselov, M. Katsnelson, F. Schedin, D. C. Elias, J. A. Jaszczak, and A. Geim, Physical review letters **100**, 016602 (2008).
